# BCR-associated factors driving chronic lymphocytic leukemia cells proliferation *ex vivo*

**DOI:** 10.1038/s41598-018-36853-8

**Published:** 2019-01-24

**Authors:** Cédric Schleiss, Wassila Ilias, Ouria Tahar, Yonca Güler, Laurent Miguet, Caroline Mayeur-Rousse, Laurent Mauvieux, Luc-Matthieu Fornecker, Elise Toussaint, Raoul Herbrecht, Frédéric Bertrand, Myriam Maumy-Bertrand, Thierry Martin, Sylvie Fournel, Philippe Georgel, Seiamak Bahram, Laurent Vallat

**Affiliations:** 1 Laboratoire d’ImmunoRhumatologie Moléculaire, INSERM UMR-S1109, LabEx Transplantex, Fédération de Médecine Translationnelle de Strasbourg (FMTS), Université de Strasbourg, Strasbourg, France; 2 Fédération Hospitalo-Universitaire (FHU) OMICARE, Université de Strasbourg, Strasbourg, France; 3 Laboratoire d’Immunologie, Plateau Technique de Biologie, Pôle de Biologie, Nouvel Hôpital Civil, Strasbourg, France; 4 Université de Strasbourg, INSERM, IRFAC UMR-S1113, Strasbourg, France; 5 Laboratoire d’Hématologie, Hôpital de Hautepierre, Hôpitaux Universitaires de Strasbourg, Strasbourg, France; 6 Service d’Hématologie Adulte, Hôpital de Hautepierre, Hôpitaux Universitaires de Strasbourg, Strasbourg, France; 7 Institut de Recherche Mathématique Avancée IRMA, CNRS UMR 7501 Strasbourg, France; 8 CNRS UPR 9021 - Immunologie et Chimie Thérapeutiques, Institut de Biologie Moléculaire et cellulaire (IBMC), Strasbourg, France; 9 CNRS UMR7199, Université de Strasbourg, Illkirch, France; 10 Present Address: Université de Strasbourg, INSERM, IRFAC UMR-S1113, and Laboratoire d’Hématologie, Hôpital de Hautepierre, Hôpitaux Universitaires de Strasbourg, Strasbourg, France

## Abstract

A chronic antigenic stimulation is believed to sustain the leukemogenic development of chronic lymphocytic leukemia (CLL) and most of lymphoproliferative malignancies developed from mature B cells. Reproducing a proliferative stimulation *ex vivo* is critical to decipher the mechanisms of leukemogenesis in these malignancies. However, functional studies of CLL cells remains limited since current *ex vivo* B cell receptor (BCR) stimulation protocols are not sufficient to induce the proliferation of these cells, pointing out the need of mandatory BCR co-factors in this process. Here, we investigated benefits of several BCR co-stimulatory molecules (IL-2, IL-4, IL-15, IL-21 and CD40 ligand) in multiple culture conditions. Our results demonstrated that BCR engagement (anti-IgM ligation) concomitant to CD40 ligand, IL-4 and IL-21 stimulation allowed CLL cells proliferation *ex vivo*. In addition, we established a proliferative advantage for ZAP70 positive CLL cells, associated to an increased phosphorylation of ZAP70/SYK and STAT6. Moreover, the use of a tri-dimensional matrix of methylcellulose and the addition of TLR9 agonists further increased this proliferative response. This *ex vivo* model of BCR stimulation with T-derived cytokines is a relevant and efficient model for functional studies of CLL as well as lymphoproliferative malignancies.

## Introduction

Like in most mature lymphoproliferative malignancies, an antigenic stimulation is believed to drive the leukemogenic process in chronic lymphocytic leukemia (CLL)^1–3^. A restricted use of *IGHV* genes and the existence of stereotypic B cell receptor (BCR) on CLL cells^4–6^ provides evidence in favor of antigenic stimulation where different microbial antigens, as well as auto-antigens, have been suspected as actors of this chronic stimulation^7^. In addition, a chronic BCR self-activation has been shown in subtypes of CLL cells^8^. Moreover, several signaling aberrations have been described downstream of the BCR, notably in aggressive CLL with unmutated *IGHV* (UM-CLL), in which the expression of ZAP70 reinforces BCR responsiveness^9–12^. BCR activation, which is essential for the physiological development of lymphocytes^13^ would also be indispensable for the survival and proliferation of CLL cells *in vivo*^2^. Accordingly, withdrawal of this stimulation is believed to be responsible for the rapid spontaneous apoptosis of CLL cells *ex vivo*^14^. The cellular consequences of this BCR activation has been extensively studied and we previously described the specific transcriptional^15,16^ and proteomic programs^17^ which are induced in aggressive CLL cells following BCR ligation.

Nevertheless, a sustained soluble stimulation of the BCR induces apoptosis in CLL cells^9,15,18–21^ and BCR-associated factors are mandatory in inducing CLL cells proliferation. Several factors, are known for their role in CLL cells survival or proliferation, among which IL-2, IL-4, IL-10, IL-15, IL-21 and CD40L are prominent^22–25^, but an exhaustive evaluation of their role as BCR-cofactors for CLL cells proliferation is still lacking. Difficulties to achieve robust CLL cell proliferation *ex vivo* led to the use of stromal cells^26,27^, activated T cells^22,28–31^ or fibroblast (eventually CD40L transfected)^21,22,30,32–34^ as feeder cells. However, feeder cells’ interactions^35^ and secretion of IL-6, IL-10 or TGF-β can also participate in CLL cells survival and proliferation^26^, which makes the identification of essential leukemogenic factors difficult and prevents the specific evaluation of BCR ligation in the proliferative response in these models.

In this study, we aim to set-up culture conditions, primarily based on BCR ligation for patho-physiological relevance, inducing CLL cells proliferation. This study was conducted in two steps. We first aimed at establishing the optimal *ex vivo* model for CLL cells proliferation measured by carboxyfluorescein succinimidyl ester (CFSE) incorporation. For this, a selection of healthy and primary CLL cells were stimulated by anti-IgM ligation with or without co-stimulatory molecules (IL-2, IL-4, IL-10, IL-21, IL-15, sCD40L), at various concentration in different culture conditions. Next, using the optimized culture conditions, we analyzed the proliferative response of fresh negatively selected B cells isolated from a cohort of well characterized CLL patients, under informed consent, including clinical data, cell morphology, flow cytometry - including ZAP70 expression status-, FISH and *IGHV* mutational status, as these factors may impact the cell response to stimulation^22,28,30,31^. These culture conditions induced a proliferative response of a fraction of CLL cells, essentially ZAP70+, in soluble medium and a proliferation of nearly all CLL cells in 3D semi-solid medium, representing a valuable system for CLL functional studies.

## Results

### Establishing culture conditions for CLL cells proliferation *ex vivo*

**Figure 1:**
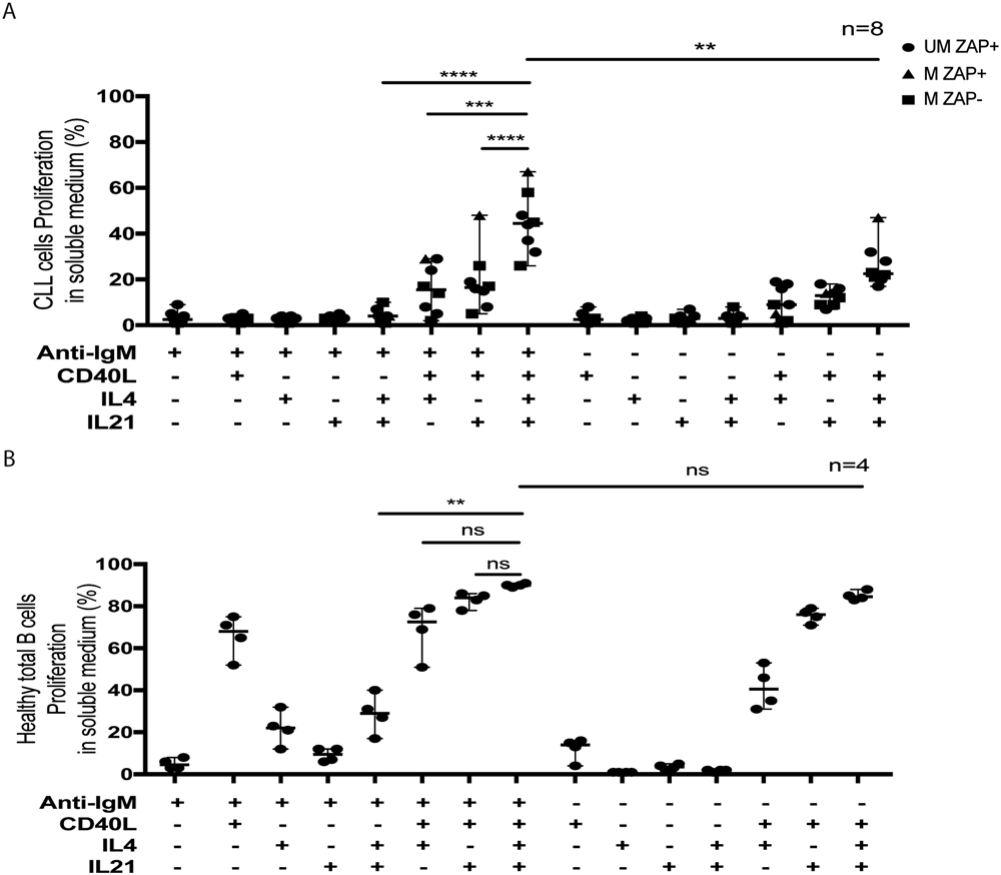
Determination of the optimal culture conditions for CLL and healthy B cells proliferation *ex vivo*. (**A**) Effect of BCR and cytokines stimulation, isolated or in combination, on the proliferation of B cells harvested from CLL patients and cultured on soluble medium (n = 8; CLL samples #6, 14, 24, 42, 49, 52, 58 and 62) and **(B)** total B cells from healthy donors (n = 4) cultured in soluble medium. After initial CFSE staining at day 0, the percentage of dividing cells (CFSE^dim^) were evaluated by flow cytometry at day 6 for CLL cells samples and at day 4 for healthy B cells. Symbols represent CLL cells sub-types (circle: UM ZAP + ; triangle: M ZAP + ; square: M ZAP-). 95% confidence interval for median is shown in each group. *p < 0.05; **p < 0.01; ***p < 0.001.

To establish culture conditions for CLL cells proliferation after *ex vivo* activation, we first evaluated CFSE labeling in a small series of patient samples (n = 8). This approach allows calculating the percentage of dividing cells and the number of cell generations (Fig. S1). We first confirmed data from previous studies showing that *ex vivo* BCR activation by means of anti-IgM ligation does not induce CLL cells proliferation when these cells are cultured in soluble medium (Figs 1A and S2A). Similarly, stimulation with IL-4, IL-21 or CD40L, used separately, in soluble medium, did not induce CLL cells proliferation either (Fig. 1A). We also confirmed that different combinations of cytokines, [CD40L + IL-4], [CD40L + IL-21] and [CD40L + IL-4 + IL-21] induced a weak (less than 40%) proliferation of CLL cells (Fig. 1A). Of note, IL-21, which has a pro-apoptotic effects on CLL cells^34^ potentiates the proliferating effect of IL-4 when sequentially added after IL-4^23^ and therefore IL-21 was added 24 h after all initial IL-4 stimulation. However, when we analyzed the proliferative effect of a combination of cytokines added after initial BCR stimulation (IgM ligation), we established that, even if BCR activation associated to [CD40L + IL-4] or [CD40L + IL-21] allowed a weak proliferation, the combination of anti-IgM with [CD40L + IL-4 + IL-21] induces a higher proliferation rate of CLL cells in soluble medium (Fig. 1A). Similar experiments confirmed the proliferative potential of these conditions on total B cells from healthy donors (Figs 1B and S2B). We analyzed the morphology of CLL cells submitted to these culture conditions. We observed the formation of clusters of proliferating cells in the culture medium (Fig. S1) and cytological analysis of these cells after cytocentrifugation at day 6 revealed in all cases a monomorphic evolution consisting in large cells with a high amount of basophilic cytoplasm, prominent nucleoli and a fine chromatin that were distinct from those of control unstimulated cells (Fig. S3A). Immunophenotyic analysis of proliferating CLL cells at day 6 after *ex vivo* stimulation showed a lower expression of CD5, an upregulation of CD138 but not of CD38 and no IgG expression on cell surface, as compared to the expression at day 0 before stimulation (Fig. S3B) which underlined the biological relevance of this model of *ex vivo* stimulation.

### Response of a cohort of CLL cells submitted to the selected culture conditions

**Figure 2:**
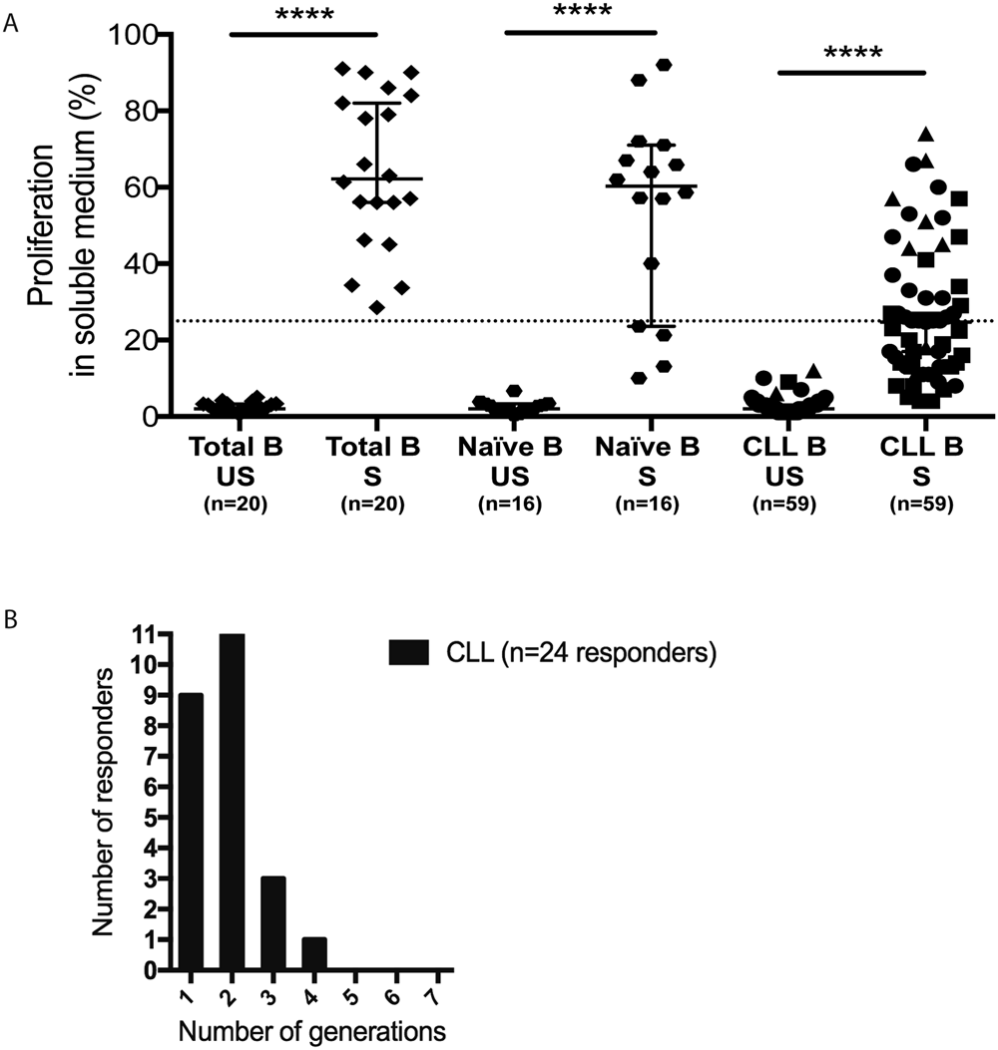
Cell proliferation of CLL and healthy B cells after soluble [anti-IgM + CD40L + IL-4 + IL-21] stimulation. (**A**) CLL cells isolated from a cohort of 59 CLL patients and healthy donors (naïve B cells, n = 16 or total B cells, n = 20) were stimulated *ex vivo* with anti-IgM, CD40L, IL-4 and IL-21. After initial CFSE staining (day 0), the percentage of cell proliferation was measured at day 6 for CLL cells and at day 4 for healthy B cells in stimulated cells (S) and control unstimulated cells (US). A threshold of dividing cells greater than 25% among living cells (dashed line) and the presence of at least one generation of daughter cells defines proliferation. 95% confidence interval for median is shown in each group. *p < 0.05; **p < 0.01; ***p < 0.001. Symbols represent CLL cells sub-types (circle: UM ZAP+; triangle: M ZAP+; square: M ZAP−; diamond: UM ZAP−). (**B**) For CLL cells responding to the stimulation, the number of cell generations, based on CFSE analysis, was quantified.

Because of the clinical and biological heterogeneity of CLL patients, we analyzed the impact of these selected culture conditions on the proliferative response of fresh CLL cells harvested from sixty-five untreated patients referred in Strasbourg Hospitals, essentially Binet stage A (58/65) (Table 1). Among them, 25 harbored unmutated *IGHV* genes (UM-CLL) and expressed the ZAP70 protein (ZAP70+), 29 had mutated *IGHV* genes (M-CLL) and did not express the ZAP70 protein (ZAP70−), 10 were ZAP70 + M-CLL and one was ZAP70− UM-CLL. We also analyzed the proliferation of total B cells (20 healthy blood-donors) and naïve (CD19^+^, CD27^−^, IgM^+^) B cells (16 healthy blood-donors). All of the total B-cells (20/20) and 12 out of 16 naïve B cells exhibited more than 25% of dividing cells at day 4 (Fig. 2A) (median 62%; confidence interval (CI) of median [56;82] with up to 4 cell generations for total B cells and median 60%; CI [24;71] for naïve B cells with up to 5 cell generations). In the same conditions, 24/59 (41%) of CLL cells samples proliferated (median 25%; CI [17;27]), showing up to four generations of proliferating cells at day 6 (Fig. 2A,B).

**Table 1:** Clinical and biological characteristics of CLL patients.

Sample	Sex	Age at diagnosis	*IGHV* status*	VH identity (%)	ZAP70 status**	CD38 ***	cytogenetic	Binet stage	Lymphocytes (G/L)
CLL-01	M	67	M	96	neg	neg	0	A	3
CLL-03	M	82	M	87	neg	neg	del13q, del17p	B	12
CLL-04	M	43	M	94	neg	neg	del13q	A	89
CLL-05	M	51	M	90	neg	neg	0	A	62
CLL-07	M	63	M	90	neg	neg	del13q	C	60
CLL-08	M	58	M	93	neg	neg	0	A	18
CLL-09	M	60	M	93	neg	neg	nd	A	9
CLL-12	F	64	M	93	neg	neg	0	A	47
CLL-15	M	57	M	91	neg	pos	0	A	43
CLL-17	M	59	M	95	neg	neg	nd	A	27
CLL-18	F	53	M	94	neg	nd	nd	A	55
CLL-27	M	77	M	92	neg	neg	del13q	A	12
CLL-30	M	46	M	92	neg	neg	del13q	C	124
CLL-31	F	68	M	93	neg	neg	del13q	A	13
CLL-34	M	73	M	91	neg	neg	0	A	35
CLL-35	F	49	M	93	neg	neg	del13q	A	10
CLL-37	M	54	M	92	neg	neg	nd	A	15
CLL-38	F	57	M	93	neg	neg	del13q	A	7
CLL-39	F	55	M	96	neg	neg	0	A	16
CLL-43	M	45	M	95	neg	nd	del13q	A	29
CLL-45	M	63	M	92	neg	neg	nd	A	12
CLL-49	M	44	M	95	neg	neg	nd	A	65
CLL-50	F	48	M	93	neg	neg	0	A	94
CLL-52	M	76	M	nd	neg	neg	0	A	44
CLL-60	F	78	M	93	neg	neg	nd	A	4
CLL-61	M	61	M	97	neg	neg	nd	A	12
CLL-62	F	61	M	98	neg	neg	del13q	A	8
CLL-63	M	44	M	93	neg	neg	del13q	A	13
CLL-64	M	70	M	91	neg	nd	nd	A	8
CLL-06	M	73	M	97	pos	neg	del13q	A	34
CLL-11	F	41	M	92	pos	neg	0	A	13
CLL-16	M	60	M	94	pos	neg	nd	A	24
CLL-20	M	57	M	93	pos	nd	0	A	10
CLL-21	M	59	M	96	pos	neg	del13q	A	6
CLL-28	M	73	M	94	pos	neg	del13q	A	11
CLL-29	F	56	M	96	pos	neg	trisom12, del11q	A	35
CLL-46	F	42	M	96	pos	nd	del13q	B	32
CLL-47	F	33	M	96	pos	neg	del13q	A	6
CLL-56	M	63	M	97	pos	pos	nd	A	9
CLL-59	F	75	UM	99	neg	pos	nd	A	8
CLL-02	M	77	UM	100	pos	nd	trisom12	B	155
CLL-10	M	60	UM	100	pos	neg	del13q	A	119
CLL-13	M	79	UM	98	pos	pos	0	A	14
CLL-14	F	70	UM	100	pos	neg	trisom12	A	18
CLL-19	F	58	UM	100	pos	neg	0	A	28
CLL-22	M	71	UM	99	pos	pos	del13q	A	7
CLL-23	M	61	UM	100	pos	pos	0	A	56
CLL-24	M	67	UM	99	pos	nd	trisom12	A	22
CLL-25	M	41	UM	99	pos	pos	del13q, del11q	B	44
CLL-26	F	66	UM	100	pos	neg	del13q	A	142
CLL-32	M	59	UM	100	pos	pos	trisom12	A	80
CLL-33	M	54	UM	100	pos	neg	del13q	A	93
CLL-36	F	55	UM	99	pos	neg	del13q	A	15
CLL-40	M	54	UM	100	pos	pos	del13q	A	66
CLL-41	F	34	UM	99	pos	neg	0	A	5
CLL-42	F	72	UM	100	pos	pos	del13q	A	11
CLL-44	F	47	UM	99	pos	neg	0	B	79
CLL-48	M	81	UM	100	pos	neg	del17p	A	10
CLL-51	F	79	UM	100	pos	neg	del13q	A	48
CLL-53	F	56	UM	100	pos	neg	del13q	A	145
CLL-54	M	60	UM	99	pos	neg	nd	A	15
CLL-55	M	72	UM	100	pos	neg	Trisom12, t(14;19)	A	10
CLL-57	F	62	UM	99	pos	neg	0	A	5
CLL-58	M	77	UM	100	pos	neg	0	A	9
CLL-65	M	60	UM	100	pos	neg	0	A	101

*≥98% of VH identity for defining unmutated (UM) CLL B cells^52^. **<7 threshold of T-cells/CLL B cells ratio of ZAP70 mean fluorescence intensity expression for defining ZAP70 positive CLL cells^49^. ***≥30% threshold for defining CD38 positive CLL. nd indicates non determined.

### 3D semi-solid matrix increases CLL cells proliferation *ex vivo*

**Figure 3:**
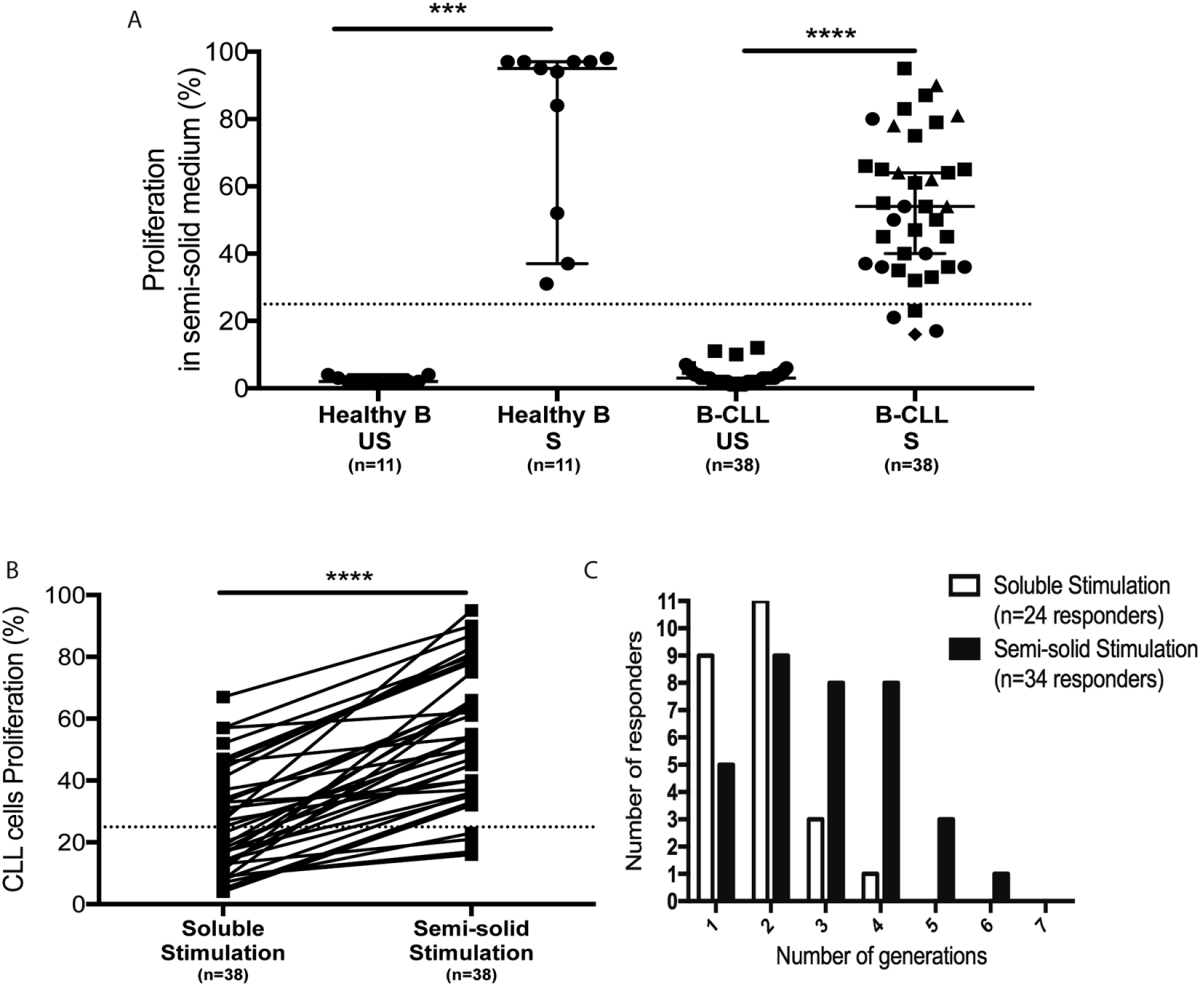
Cell proliferation of CLL and healthy B cells after [anti-IgM + CD40L + IL-4 + IL-21] stimulation on a semi-solid medium. (**A**) CLL cells isolated from a cohort of 38 CLL patients and naïve and total B cells (n = 11) isolated from healthy donors have been stimulated *ex vivo* with anti-IgM, CD40L, IL-4 and IL-21 on a semi-solid culture medium. After initial CFSE staining (day 0), the percentage of cell proliferation was measured at day 6 for CLL cells and at day 4 for healthy B cells in stimulated cells (S) and control unstimulated cells (US). *p < 0.05; **p < 0.01; ***p < 0.001. Symbols represent CLL cells sub-types (circle: UM ZAP+; triangle: M ZAP+; square: M ZAP−; diamond: UM ZAP−). **(B)** Comparison of CLL cells proliferation after anti-IgM, CD40L, IL-4 and IL-21 stimulation in soluble and semi-solid medium. **(C)** Number of cell generations for responding CLL cells stimulated in soluble and semi-solid medium.

Then we analyzed the impact of a tri-dimensional environment using a semi-solid medium (methylcellulose) where the selected culture conditions also showed their proliferative action (Fig. S4). This culture condition enhanced the proliferation rates and number of cell generations of nearly all CLL cells (89%; 34/38 samples) (Fig. 3A), with up to six generations observed at day 6 (Fig. 3B,C). Of importance, CLL cells stimulation on feeding cells (CD40L-transfected 3T6 cells) neither drove a higher proliferation rate (compared to the soluble stimulation alone) nor increased the number of generations of proliferating cells (Fig. S5A,B).

### TLR9 agonists further increases CLL cells proliferation

**Figure 4:**
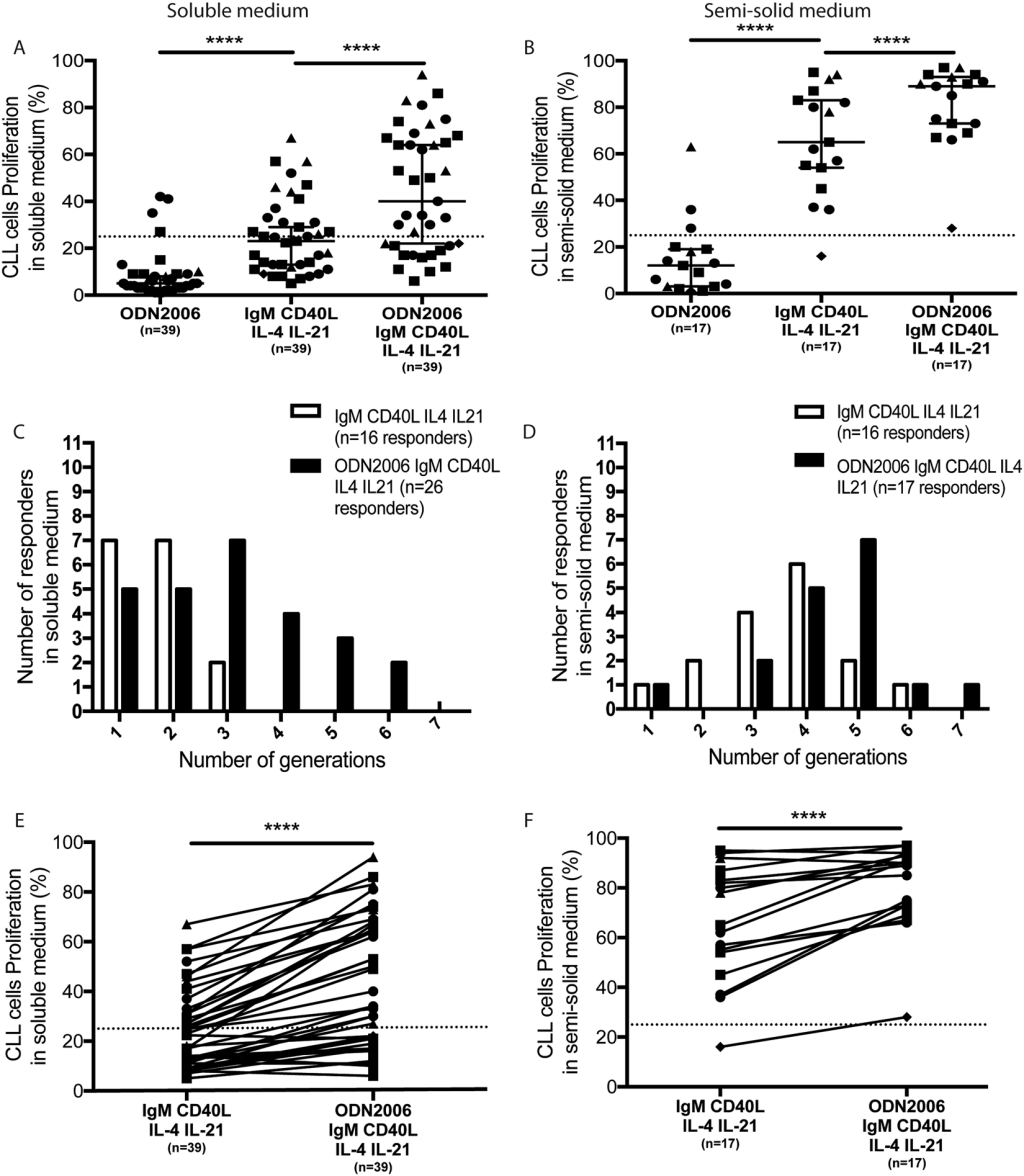
Additive effect of TLR9 activation on CLL cells after [anti-IgM + CD40L + IL-4 + IL-21] stimulation. (**A)** CLL cells (n = 39) were stimulated either by CpG-ODN2006, or IgM + CD40L + IL-4 + IL-21 or the combination of CpG-ODN2006 and anti-IgM + CD40L + IL-4 + IL-21. After initial CFSE staining (day 0), the percentage of cell proliferation was measured at day 6 in soluble medium and (**B**) in semi-solid medium. **(C)** Determination of the number of cell generations for responding CLL cells after anti-IgM + CD40L + IL-4 + IL-21 stimulation with or without CpG-ODN2006 in soluble medium and **(D)** in semi-solid medium. **(E)** Comparison of CLL cells proliferation after anti-IgM + CD40L + IL-4 + IL-21 stimulation with or without CpG-ODN2006 in soluble medium and **(F)** in semi-solid medium. Symbols represent CLL cells sub-types (circle: UM ZAP+; triangle: M ZAP+; square: M ZAP−; diamond: UM ZAP−). *p < 0.05; **p < 0.01; ***p < 0.001.

Next, we tested the effects of CpG-ODN2006 (known to affect B cells proliferation by TLR9 activation)^31^ in our culture conditions. While CpG-ODN2006 alone did not induce CLL cells proliferation (for 35 out of 39 CLL cells tested), we observed increased responses (p < 0.0001) when cells were stimulated with CpG-ODN2006 combined to BCR and cytokines in soluble and 3D semi-solid medium (Fig. 4A–F). In addition, CpG/DSP30 and IL-2 cocktails being routinely used as metaphases inductors for cytogenetic diagnosis, we then compared our stimulation conditions with CpG-ODN2006 + IL-2 or commercial premix DSP30/IL-2. The combination of CpG/DSP30 and IL-2 did not increase the proliferation rate, compared to BCR with our selected cytokine stimulation conditions, in soluble or semi-solid medium (Fig. S6A,B). Our work also confirmed previous reports^36^ showing that the combination of CpG-ODN2006 and IL-15 is a modest inducer of CLL cells proliferation. Nevertheless, IL-15 addition did not increase the proliferation rate, compared to our soluble BCR stimulation (Fig. S7A,B).

### Proliferative advantage of ZAP70+ CLL cells in soluble medium

**Figure 5:**
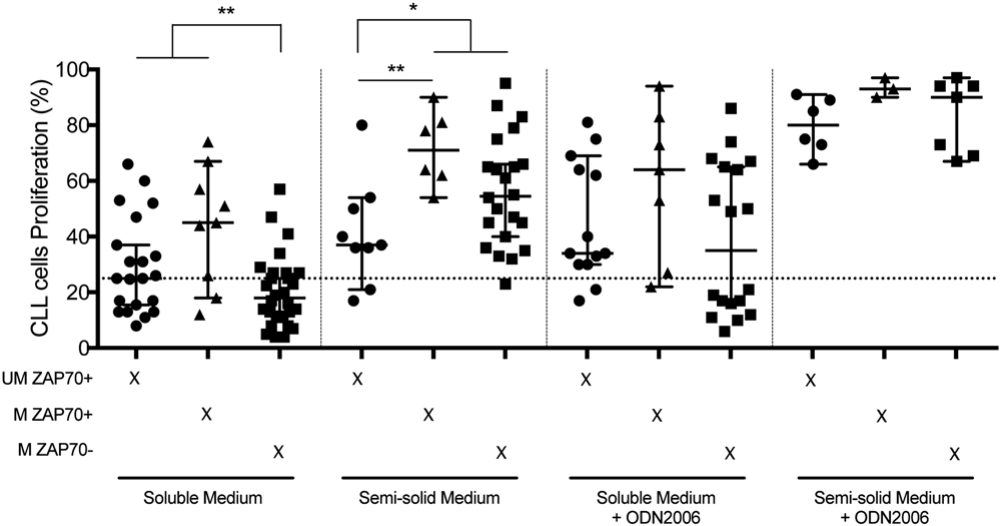
Proliferative response according to the IGHV mutational status and ZAP70 protein expression. Recapitulation of CLL cells proliferation for UM ZAP+ (circle), M ZAP70+ (triangle) and M ZAP70− (square) CLL cells after anti-IgM + CD40L + IL-4 + IL-21 stimulation in soluble medium, semi-solid medium, soluble medium + CpG-ODN2006 and semi-solid medium + CpG-ODN2006. *p < 0.05; **p < 0.01; ***p < 0.001.

Analyzing the rate of CLL cells proliferation after BCR and cytokine stimulation according to their biological characteristics, we observed that proliferating cells, in soluble medium, exhibit the highest ZAP70 expression levels, compared to non-proliferating cells (p = 0.0043) (Fig. 5). Accordingly, 15/30 (50%) ZAP70+ CLL cells responded to the stimulation with up to four generations (median: 2 generations, with a significant (p = 0.0198) Pearson’s correlation coefficient between ZAP70 expression level and the number of cell generations), whereas only 8/28 ZAP70− CLL cells proliferate with a maximum of two generations. Of note, the percentage of *IGHV* gene identity and CD38 expression did not associate with the cell proliferation in this soluble model (not shown). In semi-solid medium, ZAP70+ CLL cells with mutated *IGHV* exhibit a proliferative advantage (Fig. 5).

When BCR ligation and cytokines are associated with CpG-ODN2006 stimulation, all CLL-cells respond equally in soluble or semi-solid medium, irrespective of the level of ZAP70 and the *IGHV* status (Fig. 5).

### Increased ZAP70/SYK and STAT6 phosphorylation in proliferating ZAP70+ CLL cells

**Figure 6:**
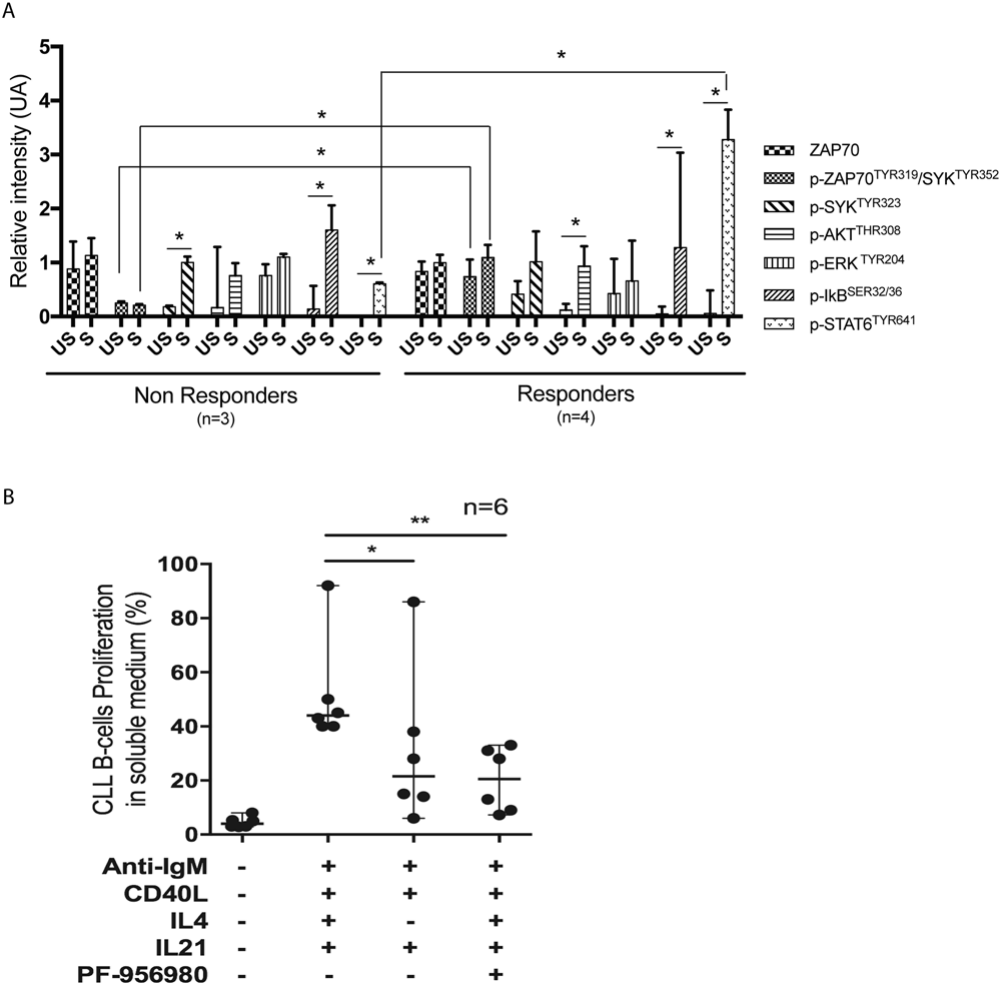
Signaling pathways activated by [anti-IgM + CD40L + IL-4 + IL-21] stimulation of responding and non-responding UM ZAP70+ CLL cells. (**A**) Normalized protein expression of ZAP70, phospho-ZAP70^Tyr319^/SYK^Tyr352^ (pZAP70/pSYK), phospho-SYK^Tyr323^ (pSYK), phospho-AKT^Thr308^ (pAKT), phospho-ERK1/2^Tyr204^ (pERK), phospho-IκB^Ser32/36^ (pIκB) and phospho-STAT6^Tyr641^ (pSTAT6), based on immunoblot results of proliferating (Responders, n = 4) and non-proliferating (Non Responders, n = 3) UM-CLL ZAP+ CLL cells following stimulation (S) or in unstimulated (US) conditions. Protein expression corresponds to the value of a signal (determined with ImageJ) normalized to that of GAPDH. (**B**) Role of IL-4 co-stimulation on CLL cells proliferation. CLL cells isolated from 6 CLL patients were stimulated *ex vivo* with anti-IgM, CD40L, IL-4 and IL-21, with or without a specific JAK3 inhibitor (PF-956980), or with anti-IgM, CD40L and IL-21. After initial CFSE staining (at day 0), the percentage of cell proliferation was measured at day 6. Symbols (circle) represent UM ZAP+ CLL cells. 95% confidence interval for median is shown in each graph. *p < 0.05; **p < 0.01; ***p < 0.001.

Given the heterogeneity of the proliferative response of ZAP70+ CLL-cells, we searched for signaling differences between responders (proliferating) and non-responders ZAP70+ cells. We performed western blots to analyze the main signaling pathways activated downstream of the BCR (e.g. ZAP70, pZAP/pSYK, pERK, pIkB) and the JAK/STAT pathway (e.g. pAKT and pSTAT6) in selected responders and non-responders amongst the ZAP70+ UM-CLL cells. Our results (Figs 6A and S8A,B) confirmed ZAP70 expression in all these CLL cells. They also suggested increased ZAP70^Tyr319^/SYK^Tyr352^ phosphorylation before stimulation (at the steady state) and a further increase upon stimulation in responding ZAP70+ CLL cells, compared to non-responding cells (p = 0.03). IkB phosphorylation was evidenced upon stimulation in both non-responding (p = 0.03) and responding (p = 0.05) CLL cells. The main signaling pathway downstream IL-21R (pSTAT3), revealed no difference between responders (proliferating) and non-responders ZAP70+ cells (Fig. S9A–C). However, we observed an increased STAT6 phosphorylation in responding CLL cells, compared to non-responders (p = 0.03). STAT6 being a major component of the IL-4 receptor signaling pathway, this result was corroborated by the significantly reduced CLL cells proliferation found in the absence of IL-4 in the stimulatory cocktails, or when a selective JAK3 inhibitor (PF-956980) was used (Fig. 6B).

## Discussion

Engagement of the BCR is a crucial event in CLL leukemogenesis, but is not sufficient to induce cell proliferation *ex vivo*, and is even known to promote B cells apoptosis^15,18^. This caveat can be prevented with coated anti-IgM stimulation, which promotes CLL cells survival^9^ but does not induce cell proliferation either. Therefore, BCR-induced CLL-cell proliferation *in vivo* likely requires additional co-stimulatory signals and proliferative properties of several soluble factors have been described in the literature. Here we performed an exhaustive study of the role of several co-stimulating factors, used solely or in combination, on top of anti-IgM stimulation, to identify mandatory factors sustaining CLL cells proliferation *ex vivo*. We evaluated CD40L (CD154), a TNF family member expressed on activated T-cells that activates the TNFRSF5 receptor on B cells and triggers several signaling pathways, including NF-kB and ERK, and participates in the survival, proliferation and differentiation of B cells^37^. IL-4, which is mainly secreted by T Follicular helper cell (TFH), activates STAT6 and participates in the activation and survival of B cells^38,39^, was also tested. Finally, we considered IL-21, which is produced by TFH, NKT and TH17 cells, for its role in the induction of JAK/STAT signaling in B cells^40^. IL-21 is known to induce apoptosis^34^, but plays also a role in CLL cells proliferation after priming by IL-4 and CD40L^22,23^, IL-21 was added at Day 1 in the culture medium. We also tested, isolated or in combination, IL-10, IL-2, IL-15, which did not show gain in BCR-induced proliferation effect (not shown) and were not considered further.

Several groups have already used soluble CD40L, Il-2, IL-4, IL-10, IL15 or IL-21, isolated or in combination, to stimulate CLL cells^22–24,30,34,39^. However, to our knowledge, this is the first study to evaluate their role as BCR co-stimulating factors in soluble conditions without the support of feeder cells. Indeed, most *ex vivo* models of CLL cells proliferation described in the literature are not consistently defined, as they used co-cultures of fibroblasts expressing CD40L, sometimes in the presence of IL-21^22^, to favor CLL cells survival^32,33^. Co-cultures involving autologous activated T-cells have also been reported^22,28^, including in association with a fibroblast layer^30^. The nature of the cytokines used suggests a T cell dependent activation, possibly delivered by follicular helper T-cells found in the proliferative centers described in secondary lymphoid organs of CLL patients^41–43^.

Our systematic analysis of BCR ligation associated to different co-stimulations enabled us to select the optimal condition combining BCR activation and co-stimulating factors [CD40L + IL-4 + IL-21] driving CLL cells proliferation. This cocktail was used to stimulate CLL cells harvested from a cohort of patients with different biological characteristics (*IGHV* UM/M, ZAP70+/− and CD38+/−) and control B cells from healthy donors. In these conditions, about 1/3 of CLL cells proliferate at day 6 after stimulation, among which ZAP70+ B cells appeared particularly responsive. The same stimulation, performed on a 3D semi-solid (methylcellulose) medium, induced the proliferation of nearly all (89%) CLL cells, with a high number of cell generation, representing an efficient model of CLL cells proliferation.

A T-dependent help for CLL cells proliferation has not yet been proven *in vivo*. In our model, T-cells-derived cytokines (CD40L, IL-4 and IL-21) enable BCR-activated CLL proliferation which reinforces this hypothesis. Furthermore, it was shown in the literature that the proliferation of CLL cells xenografted in NOD-SCID mice required concomitant TFH graft *in vivo*^44^, which also sustains this model. Of note, our results showed the importance of IL-4 and IL-21 co-stimulation (in addition to anti-IgM and CD40L) for CLL cells proliferation, whereas the sole association of anti-IgM and CD40L was sufficient to induce healthy B cells proliferation. Finally, BCR and cytokine stimulation on a tri-dimensional matrix of methylcellulose allowed the proliferation of most of the CLL cells tested, irrespectively of their biological characteristics. This result, which may reflect the natural history of leukemogenesis of CLL cells within secondary lymphoid organs may further enhance the pathophysiological relevance of our *ex vivo* model. Furthermore, the individualization of clusters of proliferating cells, distributed in this 3D matrix, could allow studying the heterogeneity of intraclonal responsiveness to various drugs in future studies.

High proliferation rate (up to 80% with 6–8 cell generations) was only observed in a subset of CLL cells. Such variability may reflect the clinical and biological heterogeneity of CLL patients, among whom those characterized by ZAP70+ CLL cells appeared particularly responsive, as also observed by others^32,45^. However, we noted that all ZAP70+ CLL cells do not equally respond to *ex vivo* stimulation, which prompted us to investigate in more details the molecular features of the responders and non-responders among this subpopulation. We first confirmed by Western blots the presence of ZAP70 protein in these cells. In CLL cells, ZAP70 activates and extends SYK^Tyr352^ phosphorylation, independently of ZAP70 phosphorylation^10,46^. This activation induces another SYK^Tyr526^ phosphorylation, leading to downstream BCR signaling. Our results, performed on a limited number of CLL cell samples (4R vs 3 NR) show that CLL cells that proliferate in soluble medium could achieved a higher initial ZAP70^Tyr319^/SYK^Tyr352^ phosphorylation, the mechanism of which remains to be investigated. We also show a concomitant increase in pSTAT6 in the proliferating ZAP70+ CLL cells, suggesting that signaling downstream of the IL-4R could participate in CLL proliferation, which reinforces the need to evaluate therapeutic agents inhibiting this pathway^39,47^. More generally, these results highlight the need to explore the functionality of multiple signaling pathways in relation to the heterogeneity of CLL patients. Indeed, as more kinases inhibitors are now available for therapeutic use, there appears to be a rational for further personalized use of these molecules.

In conclusion, this study demonstrates the relevance of the BCR activation, combined with a defined set of cytokines, to recapitulate CLL cells proliferation *ex vivo*. In addition, it highlights the potential roles of T cells in this process. The soluble and 3D culture models established here represent valuable systems for further studies aimed at characterizing the initial steps of malignant evolution of the CLL, with the ultimate goal to identify novel targets for therapeutic purpose.

## Materials and Methods

### Subjects and B cell isolation

Peripheral lymphocytes were isolated from 65 untreated CLL patients and from 36 healthy blood donors (buffy coats obtained from the Etablissement Français du Sang Grand Est, Strasbourg, France). All subjects gave written informed consent for this study, which was approved by the institutional review board of the Strasbourg University Hospitals and all experiments were performed in accordance with relevant guidelines and regulations. CLL cells were negatively selected from fresh blood samples using the RosetteSep^TM^ B cell enrichment cocktail (StemCell Technologies, Grenoble, France) and density gradient centrifugation (Ficoll^®^Paque Plus, GE Healthcare Life sciences, Velizy-Villacoublay, France). *IGHV* gene mutation status and ZAP70 expression were evaluated for each patient following established protocols^48,49^. Cytogenetic abnormalities were identified by metaphase analysis and fluorescence *in situ* hybridization (FISH) using a panel of probes as previously reported^50^. Total (CD19+) or naïve (CD19+, CD27+, IgM+) B cells were isolated from peripheral blood mononuclear cells (PBMC) of healthy blood donors using a negative selection kit (Human naïve B cell isolation kit, Human B cell isolation kit, Stemcell^TM^ Technologies, Grenoble, France) after density gradient centrifugation (Ficoll^®^Paque Plus, GE Healthcare Life sciences, Velizy-Villacoublay, France). The cell purity was then controlled by flow cytometry on a Cytomics FC500 System (Beckman-Coulter, Fullerton, CA) using CD19^+^ or CD19^+^/CD27^−^ staining (Beckman Coulter, Villepinte, France). CLL B cell purity was assessed after CD19^+^/CD5^+^ staining (Beckman Coulter, Villepinte, France) and ranged from 90% to 99% (median 97%). Cell differentiation was studied after anti-CD38 and anti-CD138 stainings (Beckman Coulter, Villepinte, France) at days 0 and 6.

### Culture conditions

Cells were cultured in RPMI 1640 Medium (Gibco, Paisley, UK) supplemented with 10% fetal calf serum (FCS) (Dutscher, Brumath, France) and 1% penicillin/streptomycin (Gibco, New York, USA), with or without methylcellulose (MethoCultTM, Stemcell^TM^ Technologies, Vancouver, Canada) at 37 °C, in an atmosphere enriched with 5% CO_2_. B cells at a density of 10^6^ cells/ml were stimulated in the absence or presence of 10 µg/ml of soluble F(ab’)2 anti-human IgM (Jackson ImmunoResearch, West Grove, PA, USA), 100 ng/ml of trimeric CD40L (Enzo Life Science, Villeurbanne, France), 10 ng/ml of IL-4 (R&D Systems-Bio-Techne, Lille, France) and 25 ng/ml of IL-21 (Invitrogen, Maryland, USA). Il-21 was added 24 h after initial stimulation with anti-IgM, CD40L and IL-4 by up/down pipetting in soluble and methyl cellulose medium. In each well, 100 µl of fresh soluble medium was added at Day 3. Other culture conditions included CpG (ODN2006, 5 µg/ml, InvivoGen, San Diego, USA), IL-15 (15 ng/ml, R&D), IL-2 (10 ng/ml, R&D) and PremixAmpliB DSP30/IL-2 (50 or 100 µg/10^6^ cells) (Amplitech, Compiegne, France). After 6 days, proliferation was assessed by flow cytometry.

Control and CLL cells were co-cultured on fibroblasts (3T6 cells) stably transfected with either a plasmid encoding human CD40L (3T6-CD40L) or mock transfected (3T6). Fibroblasts were pre-cultured overnight in 48-well plates (Dutscher, Brumath, France) at 5.10^4^ cells/well. At day 1, fibroblasts were X-ray-irradiated (30 Gy) and re-cultured overnight. At day 2, carboxyfluorescein succinimidyl ester (CFSE) labeled B cells (10^6^ cells/ml) were added to the fibroblast layer (10 CLL cells/1 fibroblast). At day 6 and after CD19 staining, B cells proliferation was evaluated by flow cytometry.

### CFSE-based proliferation assays

Freshly isolated B cells were labeled with 0.5 μM CellTrace^TM^ CFSE (ThermoFisher, Waltham, MA, USA) and incubated for 10 min at 37 °C in the dark. Washed CFSE-labeled cells were stimulated and cultured at 37 °C/5% CO_2_. Four or six days later, B cell proliferation was evidenced by a cell division-dependent decrease in CFSE staining intensity as evaluated by flow cytometry (Fig. S1). Fluorescence data were analyzed with CXP (Beckman Coulter, Fullerton, CA) and FlowJo v.8.7 (TreeStar, Ashland, USA) softwares.

### Apoptosis assay

Cell apoptosis was evaluated using FITC annexin-V Apoptosis detection kit and propidium iodide (PI) (both from BD Pharmingen, BD Bioscience, San Jose, CA, USA). Cells (10^6^) were washed in phosphate-buffered saline (PBS) and re-suspended in annexin buffer before the addition of FITC annexin-V and incubated for 20 min on ice in the dark. PI was then added for 5 min before flow cytometry analysis. DAPI (Sigma-Aldrich; Missouri, USA) was also used to analyze cell viability.

### Western blotting

After stimulation, B cells were centrifuged and cell pellets re-suspended in lysis buffer (1% Triton X-100, 20 mM Tris-HCl [pH 8], 130 mM NaCl, 10% glycerol, 2 mM EDTA, 1 mM PMSF, and protease inhibitors) for 20 minutes on ice. Lysates were centrifuged for 10 minutes at 300 g at 4 °C, and supernatants subjected to sodium dodecyl sulfate-polyacrylamide gel electrophoresis (SDS-PAGE) and transferred electrophoretically to polyvinylidene difluoride (PVDF) membranes. Membranes were then blocked using 5% milk in Tris-buffered saline (TBS; 20 mM Tris [pH 7.5], 150 mM NaCl) for 1 h at room temperature. The blots were then incubated with anti-ZAP70 (Clone E267) (Abcam, Paris, France), anti-phospho ZAP70^Tyr319^ (Abcam, Paris, France) - which recognizes also SYK^Tyr352^ phosphorylation in CLL B cells^46^ -, anti-phospho-SYK^Tyr323^ (Santa Cruz, Nanterre, France), anti-phospho-ERK1/2^Tyr204^ (clone E-4) (Santa Cruz, Nanterre, France), for 2 h at room temperature, anti-phospho-STAT6^Tyr641^ (Cell Signaling, France), anti-phospho-AKT^Thr308^ (clone D25E6) (Cell Signaling), anti-phospho-IkB^Ser32/36^ (clone 5A5) (Cell Signaling), anti-phospho-STAT3^Tyr705^ (clone EP2147Y) (GeneTex), anti-STAT3 (clone 79D7) (Cell Signaling) overnight at 4 °C, followed by incubation with horseradish peroxidase (HRP)-conjugated goat anti-mouse IgG or anti-rabbit monoclonal antibodies (1 h at 25 °C), and revealed by Electro Chemo Luminescence (ECL Plus Western blotting Detection Reagents (Amersham, Courtaboeuf, France) or SuperSignal West Femto Maximum Sensitivity substrate (Pierce, Courtaboeuf, France), according to manufacturers’ instructions. To confirm the presence of equal amounts of loaded proteins, membranes were incubated with anti-Glyceraldehyde 3-phosphate dehydrogenase (GAPDH, clone 6C5) (Merck Millipore, Guyancourt, France). Signals were visualized by chemiluminescence and processed by the Image Lab^TM^ software (BioRad, Marnes-la Coquette, France). The relative intensity of bands was measured and calculated using the Image J software (http://rsb.info.nih.gov/ij/index.html). The abundance of each protein has been normalized to GAPDH within the same sample on the same western blot.

### Statistical analyses

Statistical analyses were performed using R 3.2.4 (R Core Team, 2016, R Foundation for Statistical Computing, Vienna, Austria). Graphics were created using Graphpad Prism 7.0 (Graphpad Software; Inc, La Jolla, CA, USA). We used permutational ANOVA for repeated measurements (lmPerm R package, https://github.com/mtorchiano/lmPerm) to compare more than two groups. Posthoc tests were done using nonparametric multiple comparisons tests carried out with the nparcomp R package^51^. Differences between two groups were assessed using either nonparametric Wilcoxon matched-pairs signed rank test of nonparametric Mann-Whitney test (unpaired, when applicable). A p value of <0.05 was considered statistically significant. *p < 0.05; **p < 0.01; ***p < 0.001.

## Supplementary information

Supplementary Information
