# Supplementary material for: BCR-associated factors driving chronic lymphocytic leukemia cells proliferation ex vivo
Source: Sci Rep. 2019 Jan 24;9:701. doi: 10.1038/s41598-018-36853-8 (PMC6345919; doi:10.1038/s41598-018-36853-8)
Supplement: Supplementary file 1 — Supplementary Information [file 41598_2018_36853_MOESM1_ESM.pdf]

BCR-associated factors driving chronic lymphocytic leukemia cells proliferation ex vivo

Cedric Schleiss[1,2*], Wassila Ilias[1,2*], Ouria Tahar[1,2,3], Yonca Güler[4], Laurent Miguet[4,5], Caroline Mayeur-Rousse[4,5], Laurent Mauvieux[4,5], Luc-Matthieu Fornecker[4,6], Elise Toussaint[4,6], Raoul Herbrecht[4,6], Frederic Bertrand[2,7], Myriam Maumy-Bertrand[2,7], Thierry Martin[2,8], Sylvie Fournel[9], Philippe Georgel[1,2], Seiamak Bahram[1,2,3 **], Laurent Vallat[1, 2, 3 *** **]

[1] Laboratoire d'ImmunoRhumatologie Moléculaire, INSERM UMR-S1109, LabEx Transplantex, Fédération de Médecine Translationnelle de Strasbourg (FMTS), Université de Strasbourg, Strasbourg, France

[2] Fédération Hospitalo-Universitaire (FHU) OMICARE, Université de Strasbourg, Strasbourg, France

[3] Laboratoire d'Immunologie, Plateau Technique de Biologie, Pôle de Biologie, Nouvel Hôpital Civil, Strasbourg, France

[4] Université de Strasbourg, INSERM, IRFAC UMR-S1113, Strasbourg, France

[5] Laboratoire d'Hématologie, Hôpital de Hautepierre, Hôpitaux Universitaires de Strasbourg, Strasbourg, France

[6] Service d'Hématologie Adulte, Hôpital de Hautepierre, Hôpitaux Universitaires de Strasbourg, Strasbourg, France

[7] Institut de Recherche Mathématique Avancée IRMA, CNRS UMR 7501, Strasbourg, France

[8] CNRS UPR 9021 - Immunologie et Chimie Thérapeutiques, Institut de Biologie Moléculaire et cellulaire (IBMC), Strasbourg, France

[9] CNRS UMR7199, Université de Strasbourg, Illkirch, France

* equal contributions.

** corresponding authors: Seiamak Bahram siamak@unistra.fr; Laurent Vallat vallat@unistra.fr

*** current address: Université de Strasbourg, INSERM, IRFAC UMR-S1113, and Laboratoire d'Hématologie, Hôpital de Hautepierre, Hôpitaux Universitaires de Strasbourg, France

Supplemental figures

Figure S1

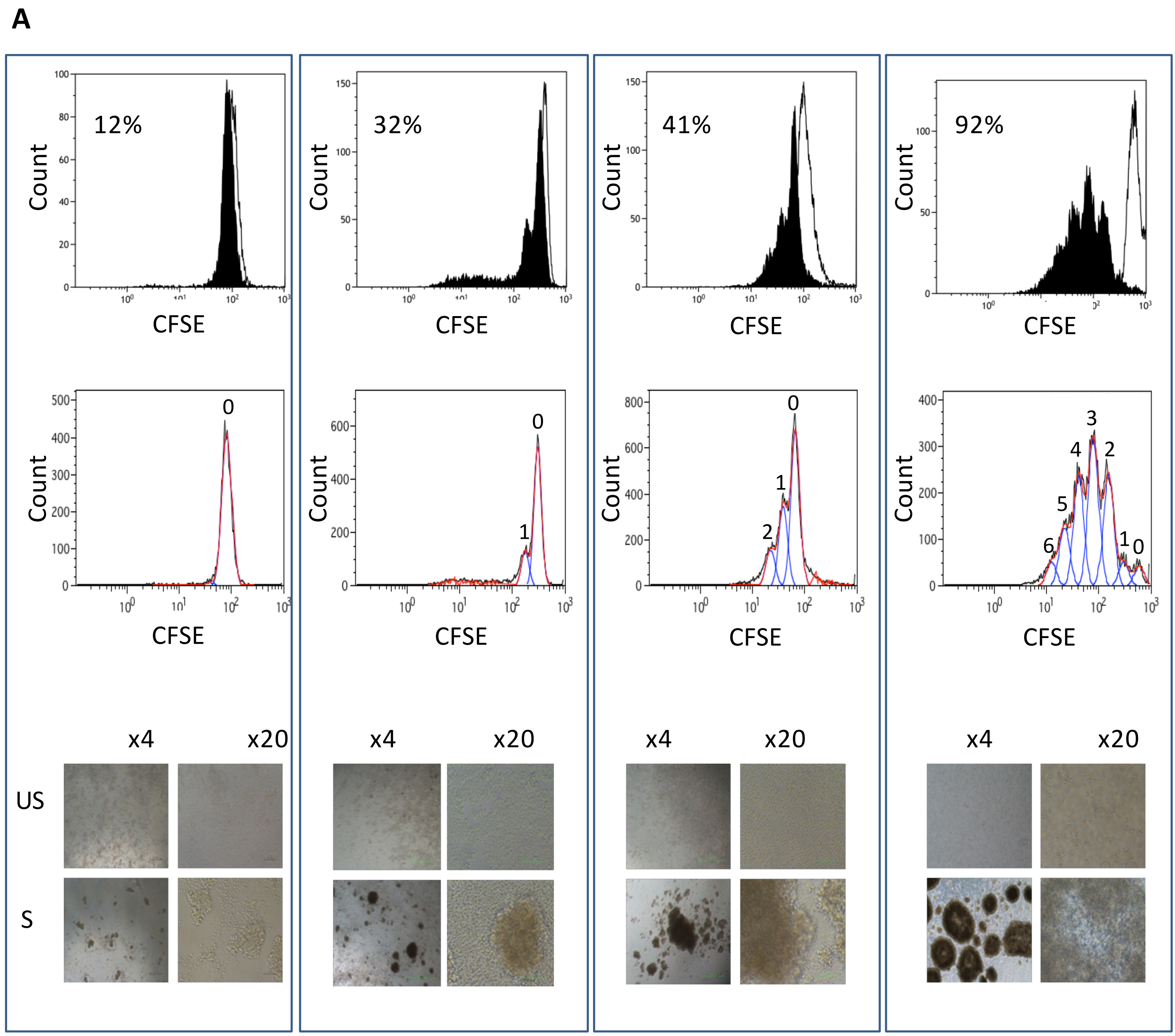

A
12%
32%
41%
92%
Count
CFSE
x4
x20
US
S

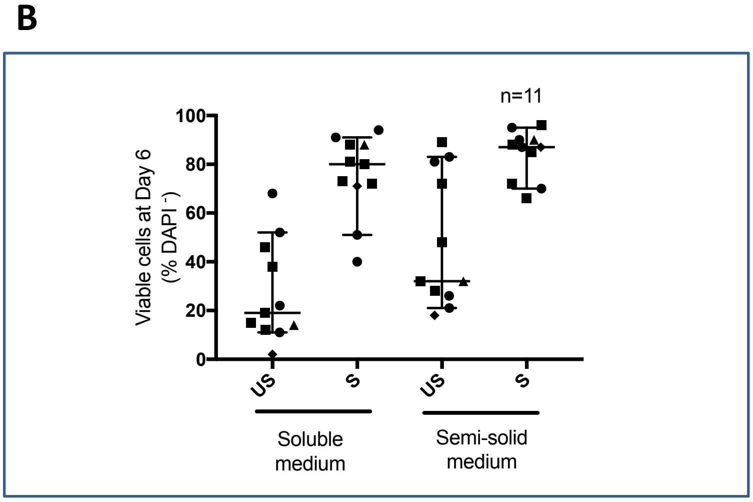

B
n=11
Viable cells at Day 6 (% DAPI⁻)
US
S
Soluble medium
Semi-solid medium

Figure S2

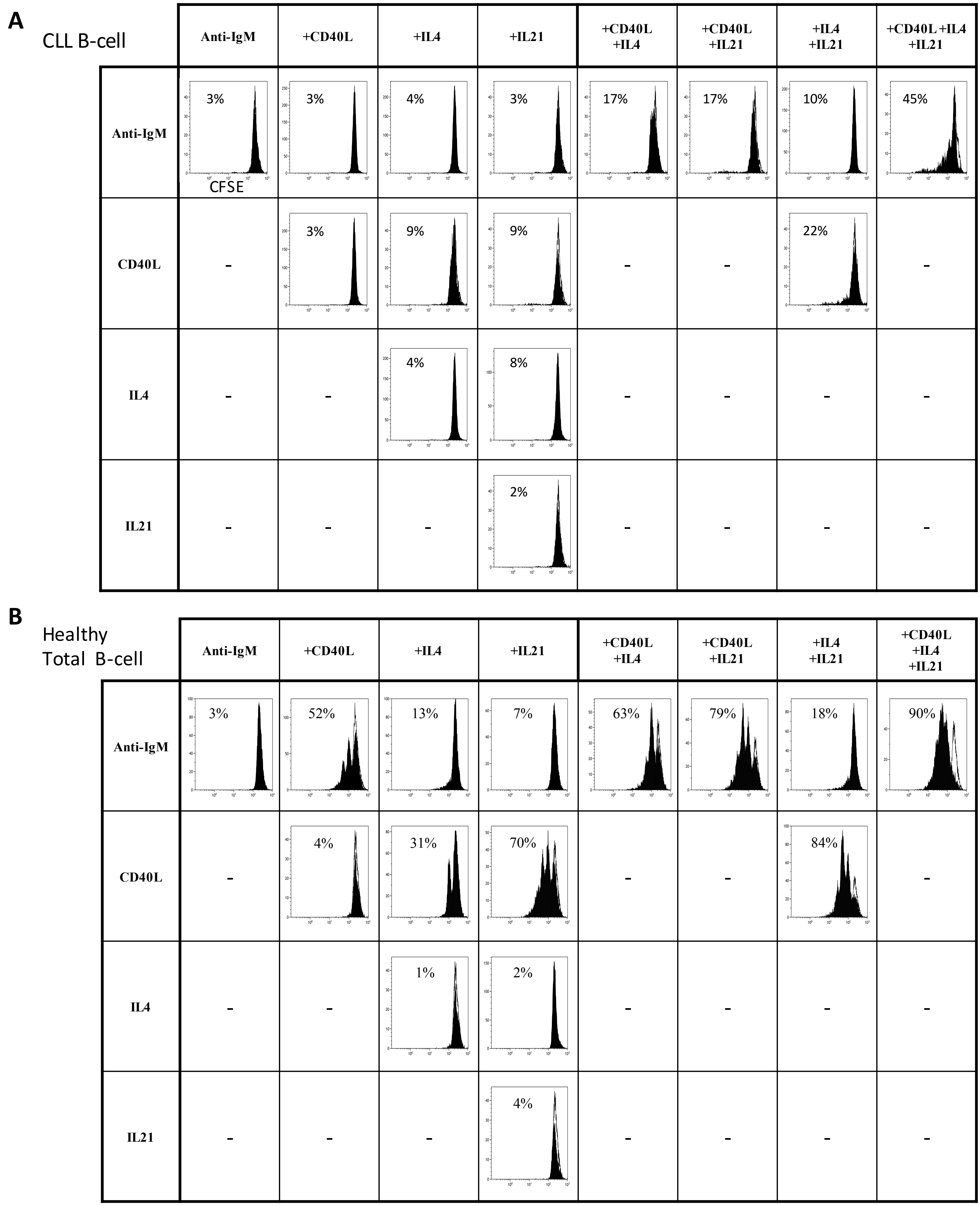
A
CLL B-cell
Anti-IgM
+CD40L
+IL4
+IL21
+CD40L +IL4
+CD40L +IL21
+IL4 +IL21
+CD40L +IL4 +IL21
Anti-IgM
3%
CFSE
3%
4%
3%
17%
17%
10%
45%
CD40L
-
3%
9%
9%
-
-
22%
-
IL4
-
-
4%
8%
-
-
-
-
IL21
-
-
-
2%
-
-
-
-
B
Healthy Total B-cell
Anti-IgM
+CD40L
+IL4
+IL21
+CD40L +IL4
+CD40L +IL21
+IL4 +IL21
+CD40L +IL4 +IL21
Anti-IgM
3%
52%
13%
7%
63%
79%
18%
90%
CD40L
-
4%
31%
70%
-
-
84%
-
IL4
-
-
1%
2%
-
-
-
-
IL21
-
-
-
4%
-
-
-
-

Figure S3

A

Day 6 control unstimulated | Day 6 stimulated

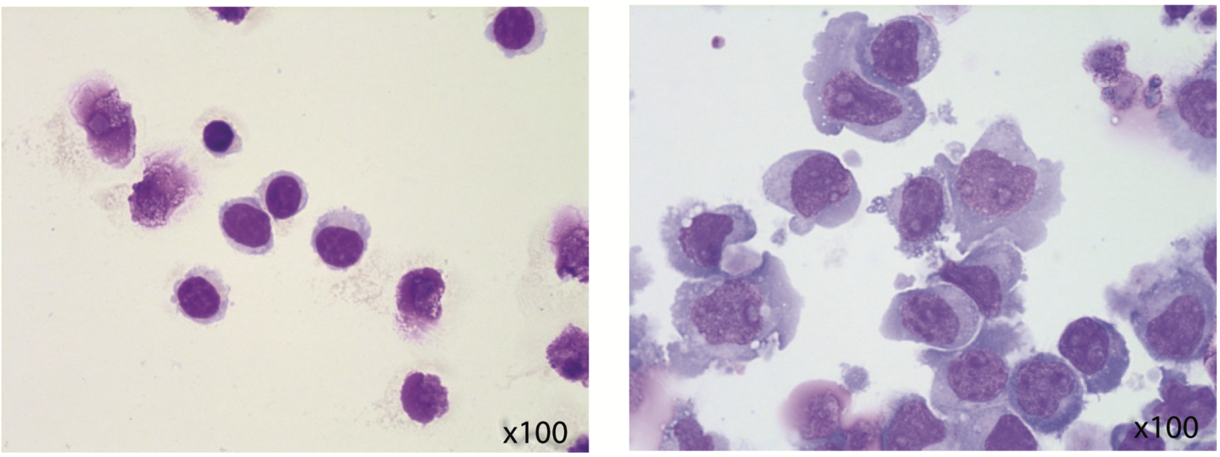

B

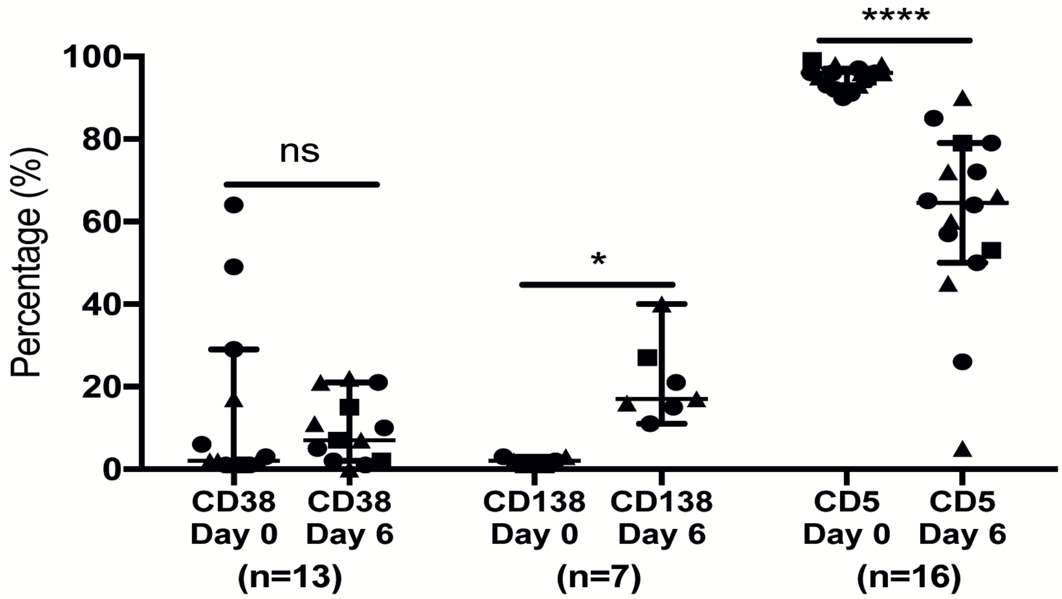

Figure S4

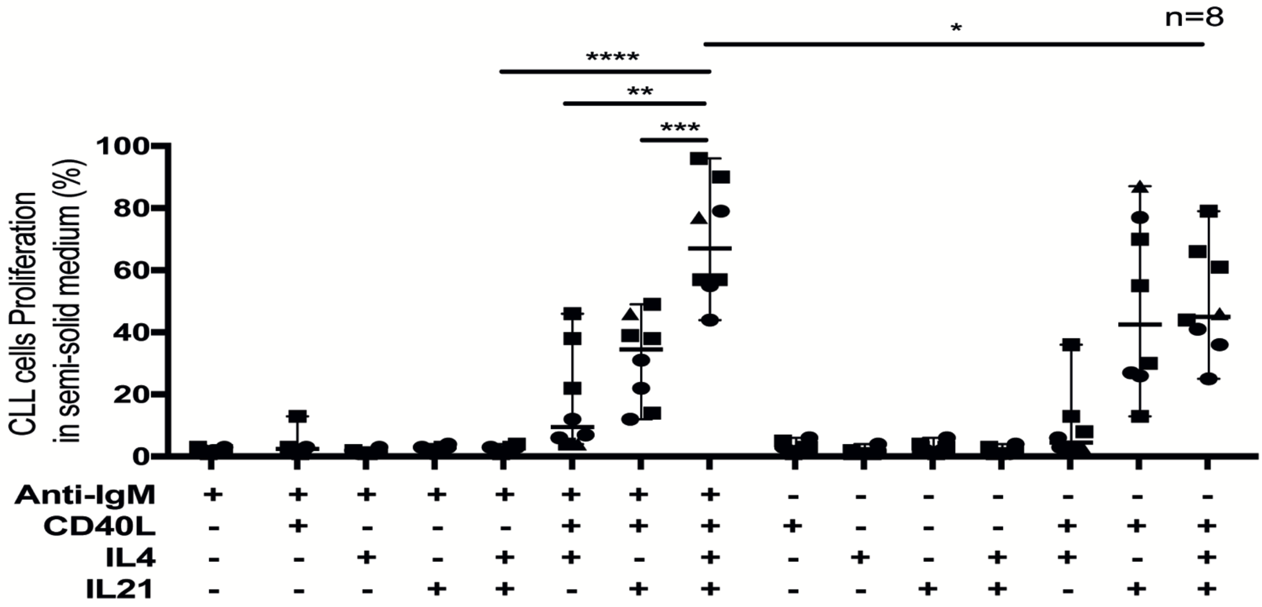

n=8
*
****
**
***
CLL cells Proliferation in semi-solid medium (%)
100
80
60
40
20
0
Anti-IgM + + + + + + + + - - - - - - -
CD40L - + - - - + + + + - - - + + +
IL4 - - + - + + - + - + - + + - +
IL21 - - - + + - + + - - + + - + +

Figure S5

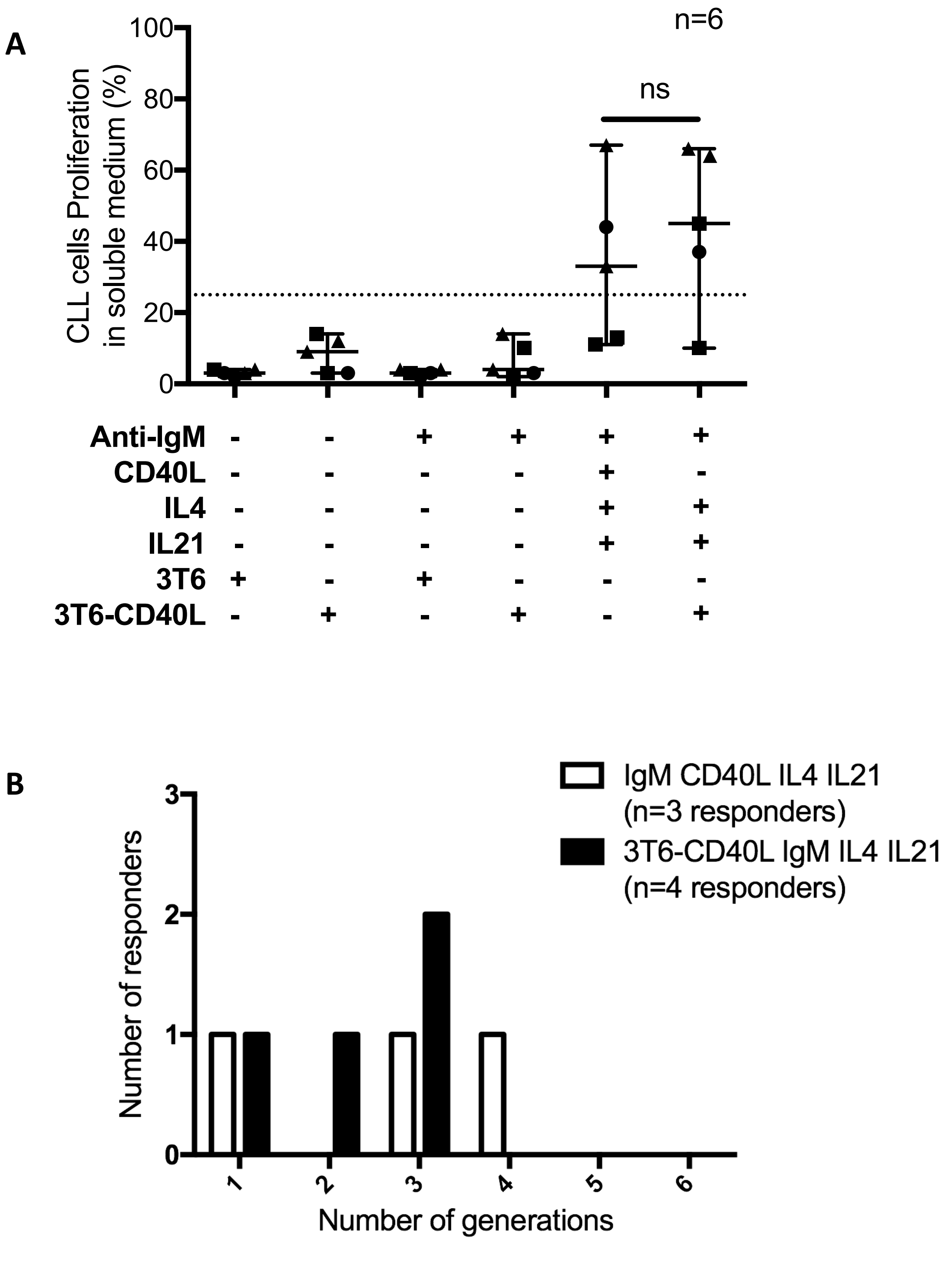
A
n=6
100
80
60
40
20
0
CLL cells Proliferation in soluble medium (%)
ns
Anti-IgM - - + + + +
CD40L - - - - + -
IL4 - - - - + +
IL21 - - - - + +
3T6 + - + - - -
3T6-CD40L - + - + - +
B
IgM CD40L IL4 IL21 (n=3 responders)
3T6-CD40L IgM IL4 IL21 (n=4 responders)
3
2
1
0
Number of responders
1
2
3
4
5
6
Number of generations

Figure S6

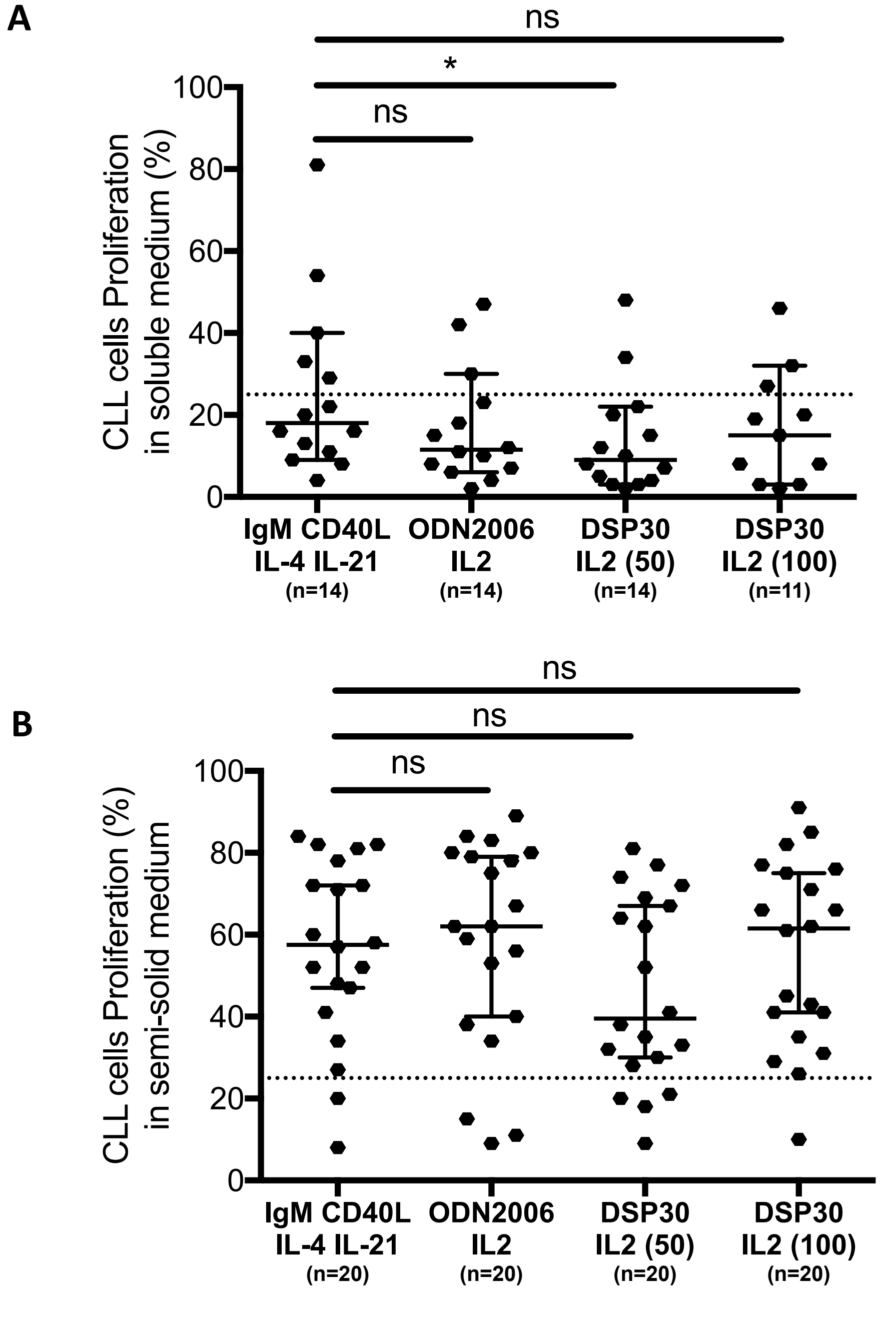
A
ns
*
ns
CLL cells Proliferation in soluble medium (%)
100
80
60
40
20
0
IgM CD40L IL-4 IL-21 (n=14)
ODN2006 IL2 (n=14)
DSP30 IL2 (50) (n=14)
DSP30 IL2 (100) (n=11)
B
ns
ns
ns
CLL cells Proliferation (%) in semi-solid medium
100
80
60
40
20
0
IgM CD40L IL-4 IL-21 (n=20)
ODN2006 IL2 (n=20)
DSP30 IL2 (50) (n=20)
DSP30 IL2 (100) (n=20)

Figure S7

A

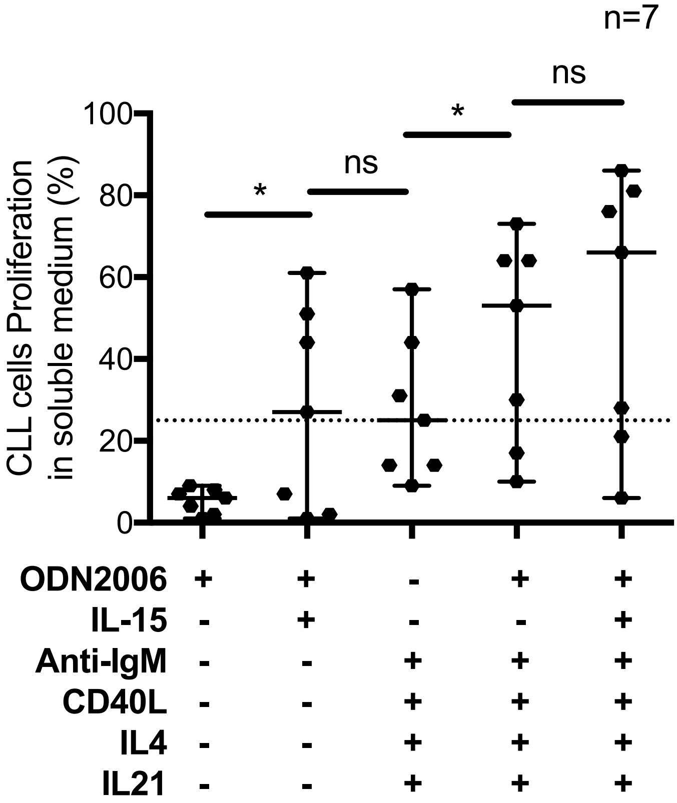

n=7
ns
*
ns
*
100
80
60
40
20
0
CLL cells Proliferation in soluble medium (%)
ODN2006 + + - + +
IL-15 - + - - +
Anti-IgM - - + + +
CD40L - - + + +
IL4 - - + + +
IL21 - - + + +

B

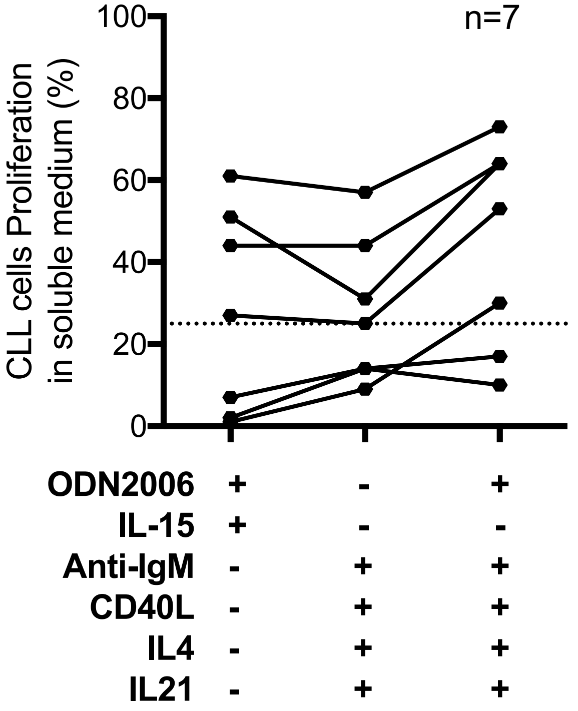

n=7
100
80
60
40
20
0
CLL cells Proliferation in soluble medium (%)
ODN2006 + - +
IL-15 + - -
Anti-IgM - + +
CD40L - + +
IL4 - + +
IL21 - + +

Figure S8 A

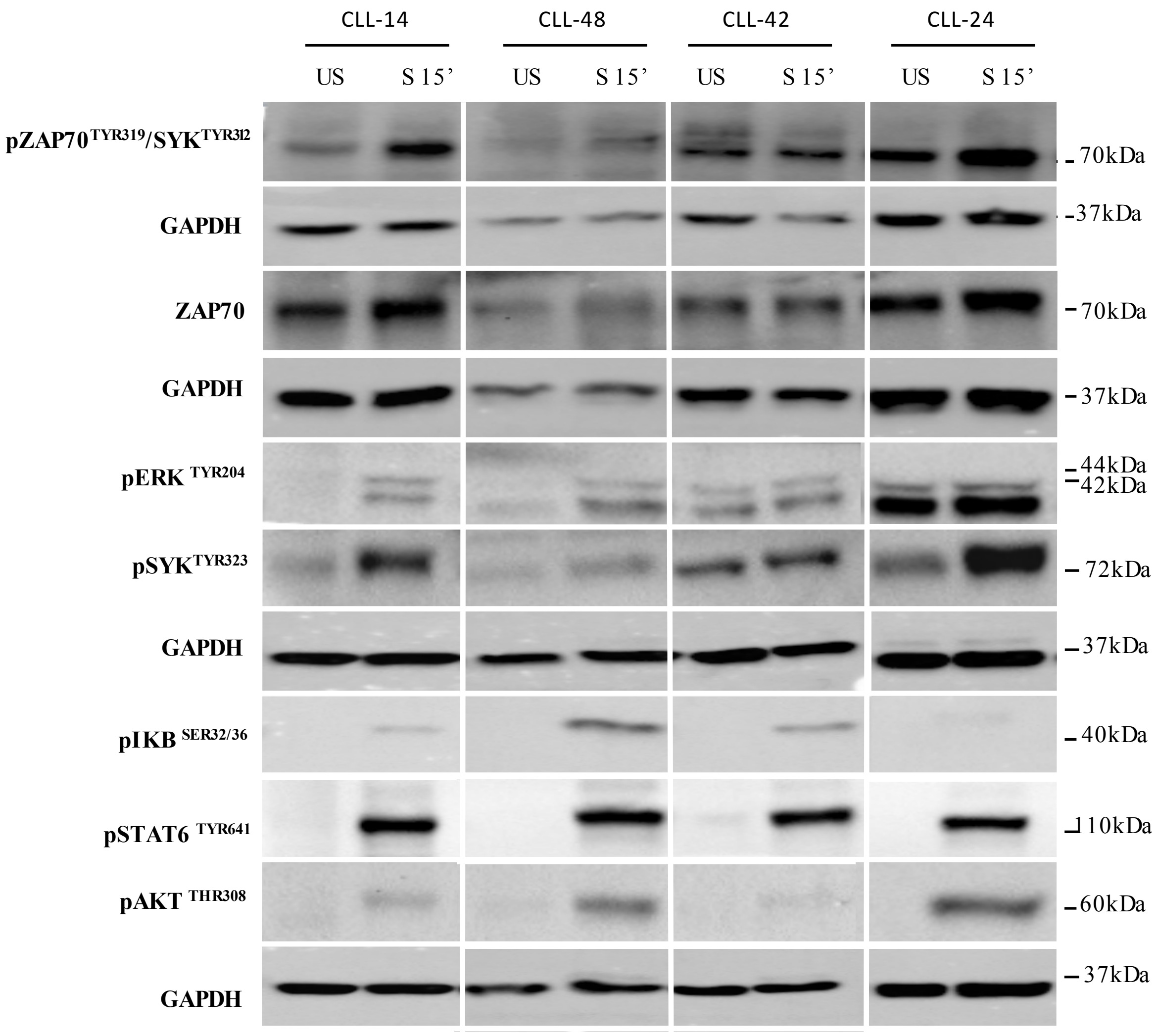
CLL-14
CLL-48
CLL-42
CLL-24
US
S 15'
pZAP70TYR319/SYKTYR312
GAPDH
ZAP70
GAPDH
pERK TYR204
pSYKTYR323
GAPDH
pIKB SER32/36
pSTAT6 TYR641
pAKT THR308
GAPDH
70kDa
37kDa
70kDa
37kDa
44kDa
42kDa
72kDa
37kDa
40kDa
110kDa
60kDa
37kDa

Figure S8 B

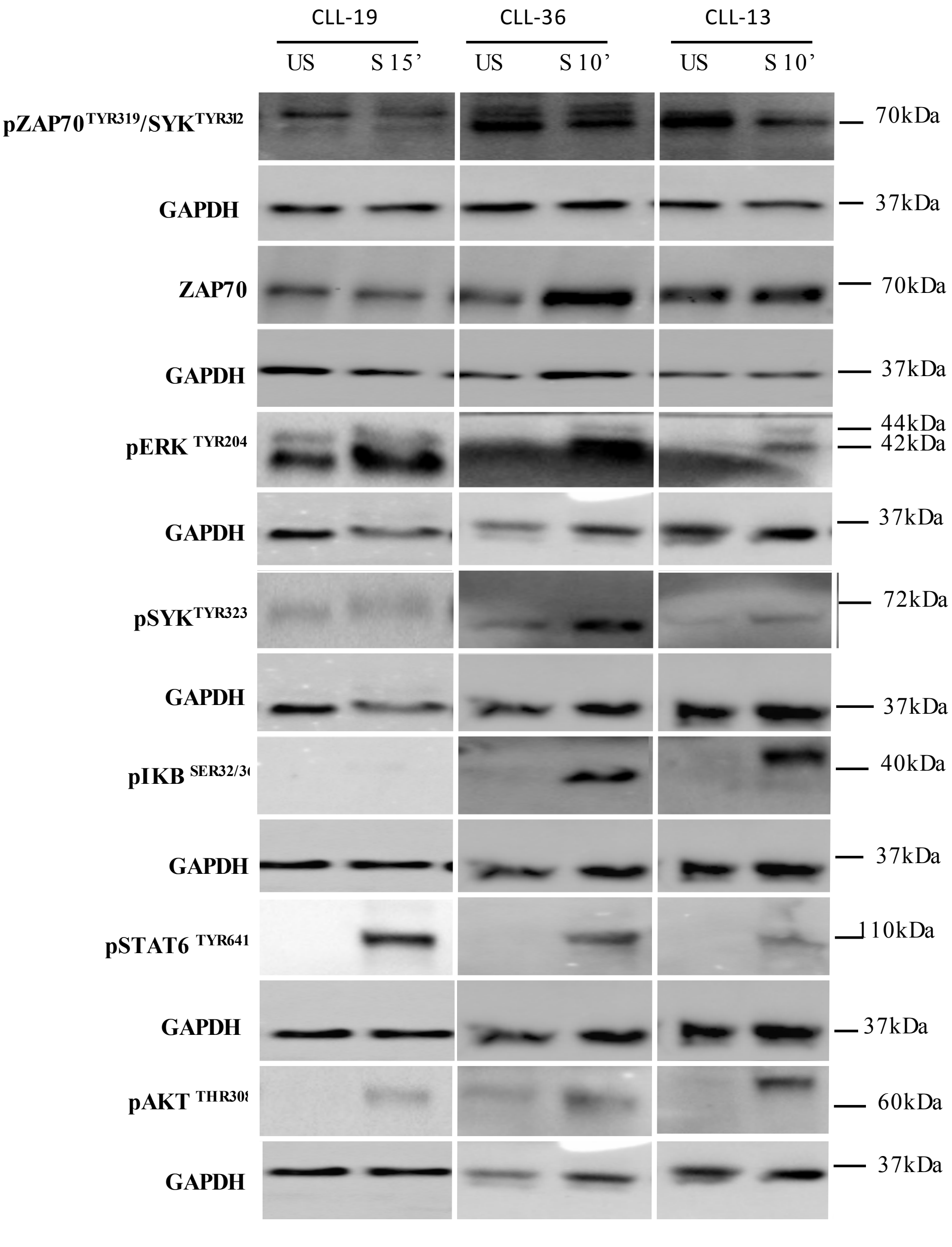

CLL-19
CLL-36
CLL-13
US
S 15'
US
S 10'
US
S 10'
pZAP70TYR319/SYKTYR352
GAPDH
ZAP70
GAPDH
pERK TYR204
GAPDH
pSYKTYR323
GAPDH
pIKB SER32/36
GAPDH
pSTAT6 TYR641
GAPDH
pAKT THR308
GAPDH
70kDa
37kDa
70kDa
37kDa
44kDa
42kDa
37kDa
72kDa
37kDa
40kDa
37kDa
110kDa
37kDa
60kDa
37kDa

Figure S9

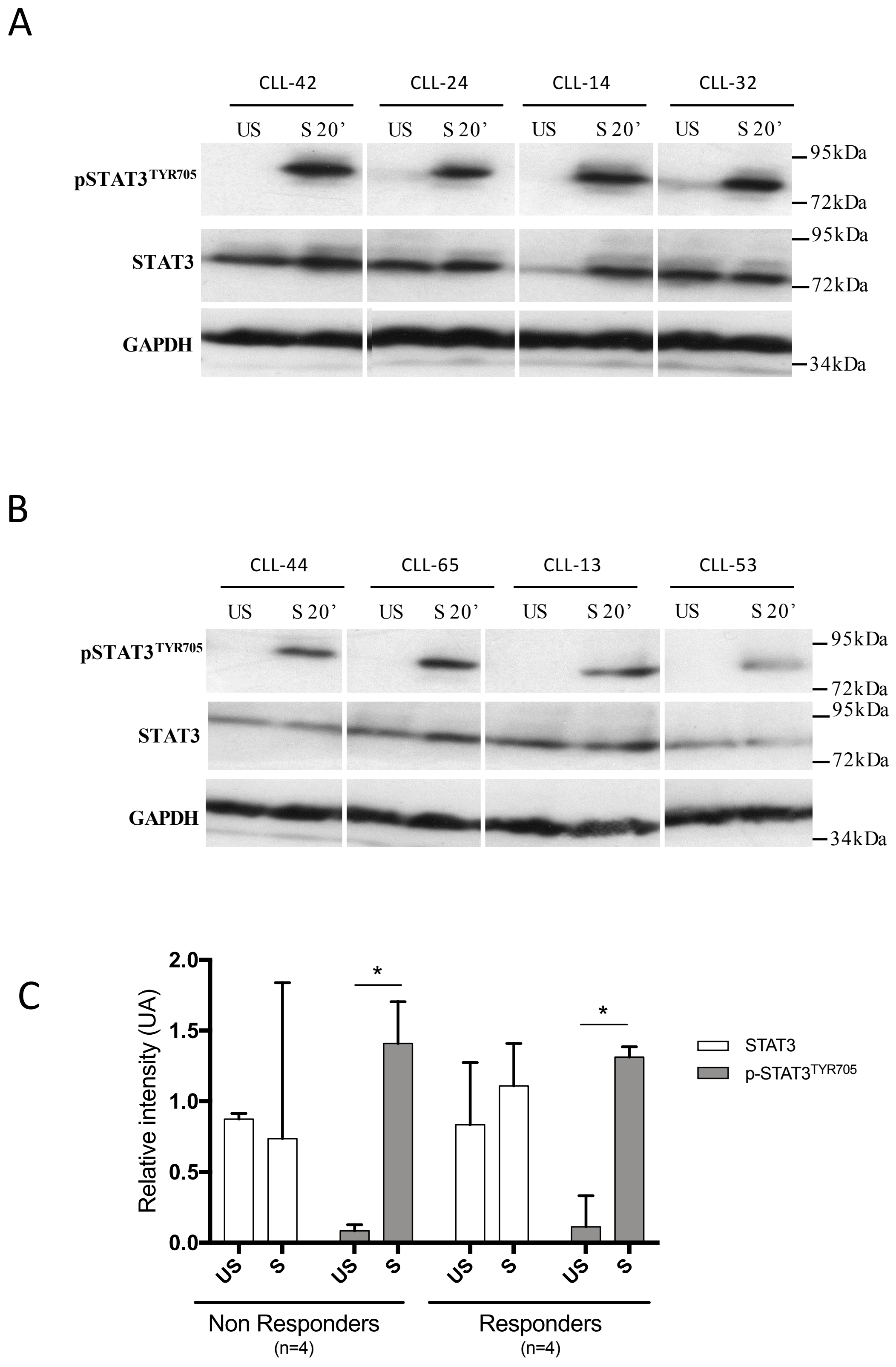
A
CLL-42
CLL-24
CLL-14
CLL-32
US
S 20'
pSTAT3^{TYR705}
STAT3
GAPDH
95kDa
72kDa
34kDa
B
CLL-44
CLL-65
CLL-13
CLL-53
C
Relative intensity (UA)
2.0
1.5
1.0
0.5
0.0
*
STAT3
p-STAT3^{TYR705}
US
S
Non Responders
(n=4)
Responders
(n=4)

Supplemental figure legends

Figure S1: Proliferation of CLL cells at day 6 after [anti-IgM + CD40L + IL-4 + IL-21] stimulation. (A) CLL cells are stimulated after initial CFSE staining at day 0. At day 6, flow cytometry analysis shows a repartition of the initial CFSE staining in the daughter cells populations, which permits the quantification of the number of generations. From left to right, representative examples of non-responding (non-proliferating), and responding (proliferating) CLL cell samples with 1, 2 or 6 generations at day 6. For each case, cell generations numbering is detailed. The pictures below show clusters of proliferating cells (magnifications x4 and x20) for stimulated (S) in lower line, and control unstimulated (US) CLL cells in upper line, at day 6 after stimulation. **(B)** Percentage of viable cells analyzed by flow cytometry after DAPI staining at day 6 in stimulated (S) and control unstimulated (US) CLL cells in soluble and semi-solid medium. Symbols represent CLL cells sub-types (circle: UM ZAP+; triangle: M ZAP+; square: M ZAP-).

Figure S2: Determination of the optimal culture conditions inducing CLL cells proliferation. (A) Different combinations of soluble culture conditions were tested on CLL and **(B)** healthy B cells in order to establish optimal culture conditions. Identical samples were subjected to all the conditions. Proliferation is measured at day 6 after initial CFSE staining at day 0. The percentage of dividing cells is indicated for each condition for representative CLL and healthy cells samples.

Figure S3: Morphology of CLL cells after soluble [anti-IgM + CD40L + IL-4 + IL-21] stimulation. (A) A cytologic analysis, after cytocentrifugation, of control unstimulated cells and stimulated cells (at day 6) are shown for a representative CLL proliferating (responders) CLL

cells (magnification x100). **(B)** Flow cytometry analysis of CLL cells before and after combined soluble anti-IgM, CD40L, IL-4 and IL-21 stimulation. Percentage of CD38, CD138 and CD5 positive cells at day 6 after *ex vivo* stimulation in proliferating (responders) CLL cells are shown. 95% confidence interval for median is shown in each graph. * $p<0.05$; ** $p<0.01$; *** $p<0.001$.

Figure S4: Proliferative response of CLL cells in semi-solid medium *ex vivo.* Effect of BCR and cytokines stimulation, isolated or in combination, on the proliferation of cells harvested from CLL patients and cultured in semi-solid medium (n=8; CLL samples # 20, 14, 24, 42, 49, 50, 52 and 62). After initial CFSE staining at day 0, the percentage of dividing cells (CFSE ^{dim}) were evaluated by flow cytometry at day 6. 95% confidence interval for median is shown in each group. * $p<0.05$; ** $p<0.01$; *** $p<0.001$.

Figure S5: Effect of 3T6-CD40L transfected cells on CLL cells proliferation.

(A) Cells isolated from CLL patients (n=6) were stimulated either with anti-IgM, or IL4 + IL-21, or anti-IgM + IL-4 + IL-21, in the presence of soluble CD40L and cocultured with 3T6 cells or 3T6-CD40L transfected cells. 95% confidence interval for median is shown in each group. **(B)** For the responding (proliferating) CLL cells, the number of cell generations after anti-IgM + CD40L + IL-4 + IL-21 or anti-IgM + 3T6-CD40L + IL-4 + IL-21 stimulation was evaluated.

Figure S6: Comparison of TLR9 + IL-2 and anti-IgM + CD40L + IL-4 + IL-21 stimulation on CLL cells. (A) CLL cells were stimulated either with IgM + CD40L + IL-4 + IL-21 or CpG-ODN2006 + IL-2 or Premix AmpliB DSP30 + IL-2 (50µl or 100µl/10^6 cells). After initial CFSE staining (day 0), the percentage of cell proliferation (CFSE^{dim}) was measured at day 6 in soluble medium, and **(B)** in semi-solid medium. 95% confidence interval for median is shown in each group. * $p<0.05$.

Figure S7. IL-15 additive effect on CLL cells proliferation. (A) Selected CLL cells were stimulated ex vivo with CpG-ODN2006, or CpG-ODN2006 + IL-15, or anti-IgM + CD40L + IL-4 + IL-21, or a combination of CpG-ODN2006 + anti-IgM + CD40L + IL-4 + IL-21 with or without IL-15. Cell proliferation was measured at day 6 after initial (day 0) CFSE staining. 95% confidence interval for median is shown in each group. * $p<0.05$. **(B)** CLL cells proliferation for 6 samples stimulated by various combinations: CpG-ODN2006 + IL-15, anti-IgM + CD40L + IL-4 + IL-21 or CpG-ODN2006 + anti-IgM + CD40L + IL-4 + IL-21.

Figure S8: Signaling pathways activated by [anti-IgM + CD40L + IL-4] stimulation. (A) Representative Western blot showing expression of ZAP70, phospho-ZAP70/SYK, phospho-SYK, phospho-AKT, phospho-ERK, phospho-IKB, phospho-STAT6 and GAPDH (as loading control), before (unstimulated) and 15 minutes after anti-IgM + CD40L + IL-4 + IL-21 stimulation for proliferating (n=4) and **(B)** non-proliferating (n=3) UM-CLL ZAP+ CLL cells.

Figure S9: Signaling pathway activated by [anti-IgM + CD40L + IL-4 + IL-21] stimulation. (A) Western blot showing expression of STAT3, phospho-STAT3 and GAPDH (as loading control), before (unstimulated) and 20 minutes after IL-21 addition at day 1 (following initial anti-IgM + CD40L + IL-4 stimulation at day 0) for proliferating (n=4) and **(B)** non-proliferating (n=4) UM-CLL ZAP+ CLL cells. **(C)** STAT3 and p-STAT3 protein expression normalized to that of GAPDH (determined with ImageJ). 95% confidence interval for median is shown in each group. * $p<0.05$.

Supplementary full-length blots for reviewing purpose.

Figure S8 A

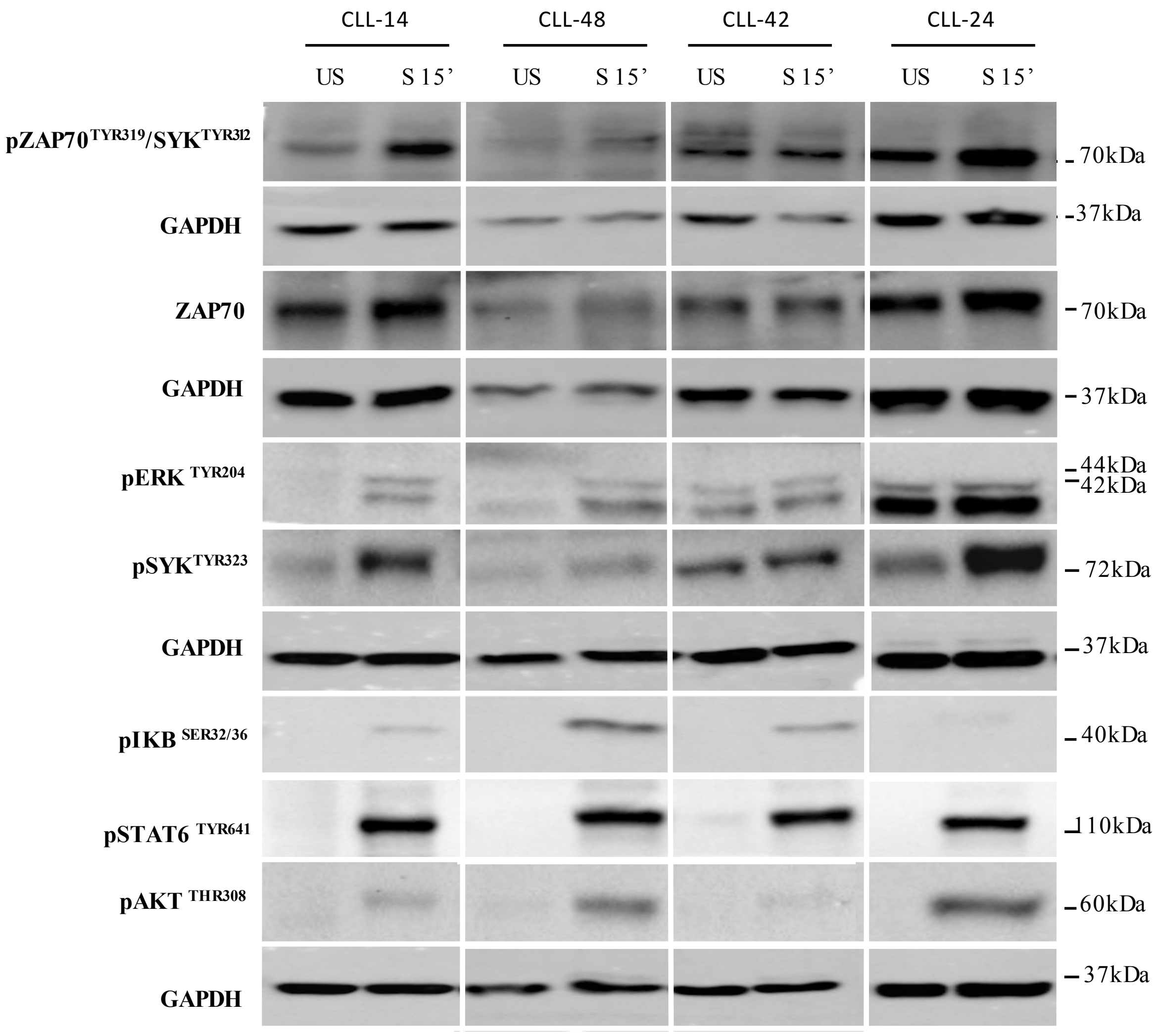
CLL-14
CLL-48
CLL-42
CLL-24
US
S 15'
pZAP70 TYR319/SYK TYR312
GAPDH
ZAP70
GAPDH
pERK TYR204
pSYK TYR323
GAPDH
pIKB SER32/36
pSTAT6 TYR641
pAKT THR308
GAPDH
70kDa
37kDa
70kDa
37kDa
44kDa
42kDa
72kDa
37kDa
40kDa
110kDa
60kDa
37kDa

Figure S8 A

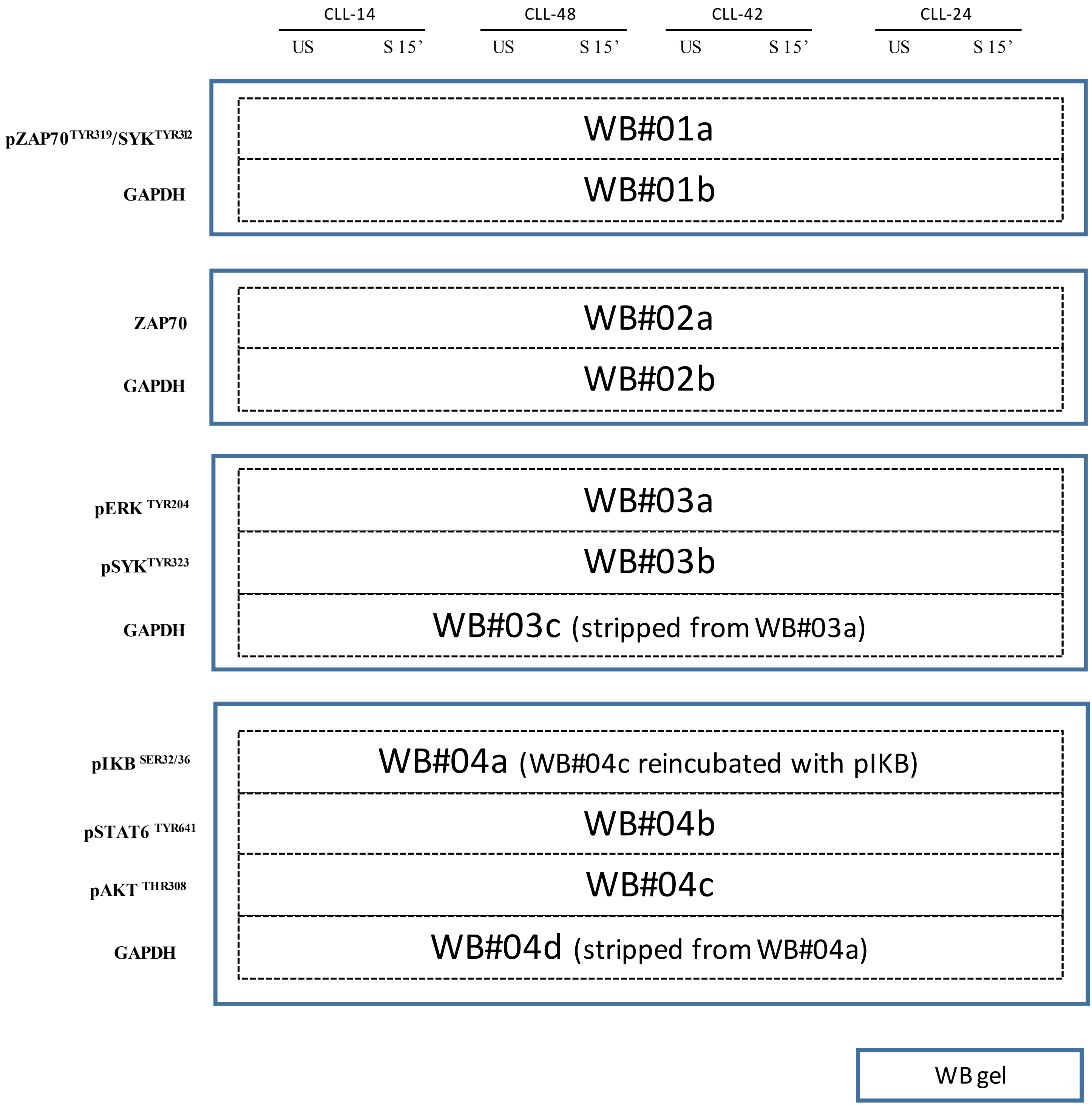
CLL-14
CLL-48
CLL-42
CLL-24
US
S 15’
pZAP70 TYR319/SYK TYR312
GAPDH
WB#01a
WB#01b
ZAP70
GAPDH
WB#02a
WB#02b
pERK TYR204
pSYK TYR323
GAPDH
WB#03a
WB#03b
WB#03c (stripped from WB#03a)
pIKB SER32/36
pSTAT6 TYR641
pAKT THR308
GAPDH
WB#04a (WB#04c reincubated with pIKB)
WB#04b
WB#04c
WB#04d (stripped from WB#04a)
WB gel

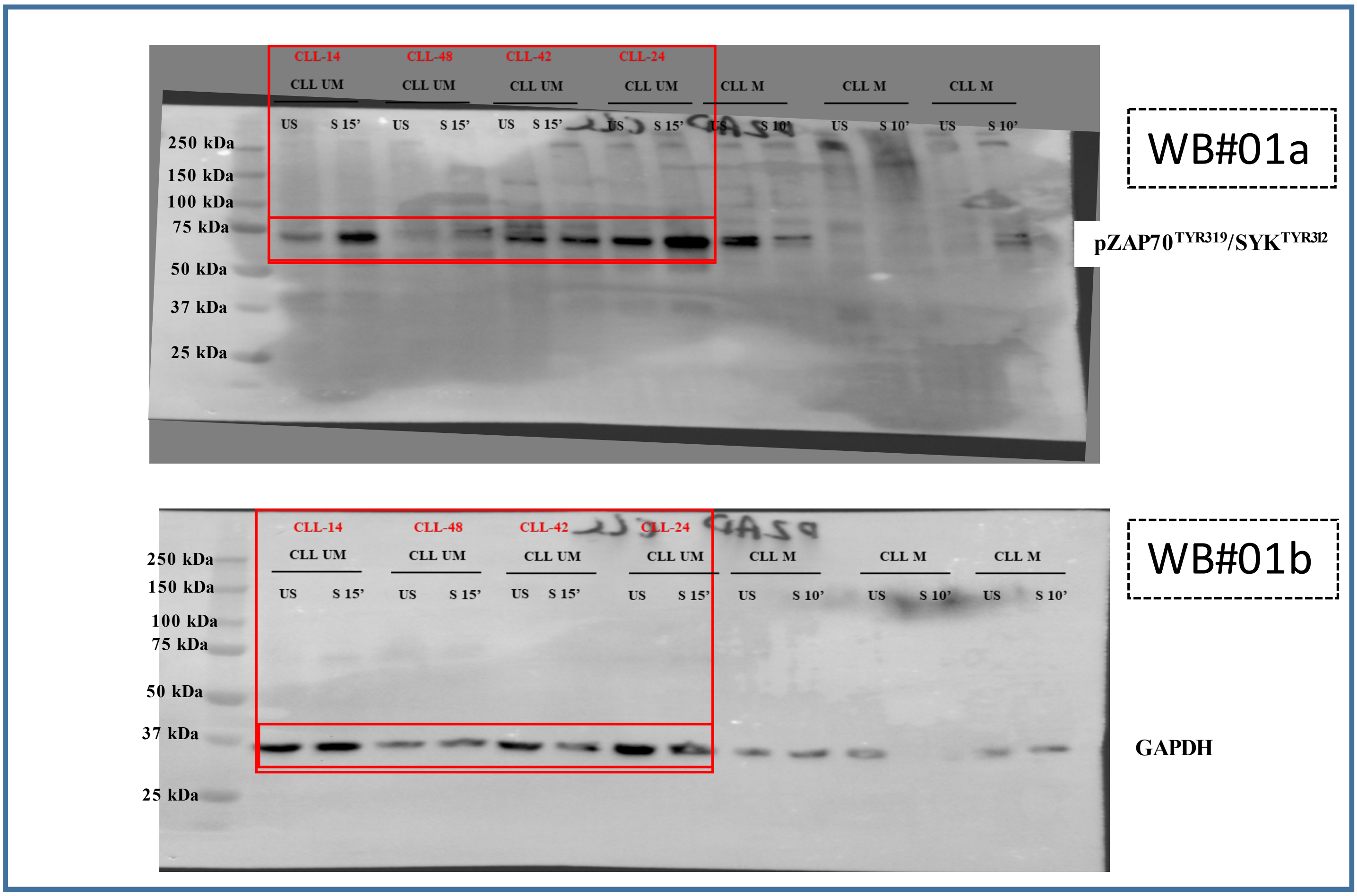
CLL-14
CLL-48
CLL-42
CLL-24
CLL UM
CLL M
US
S 15'
S 10'
250 kDa
150 kDa
100 kDa
75 kDa
50 kDa
37 kDa
25 kDa
WB#01a
pZAP70TYR319/SYKTYR312
WB#01b
GAPDH

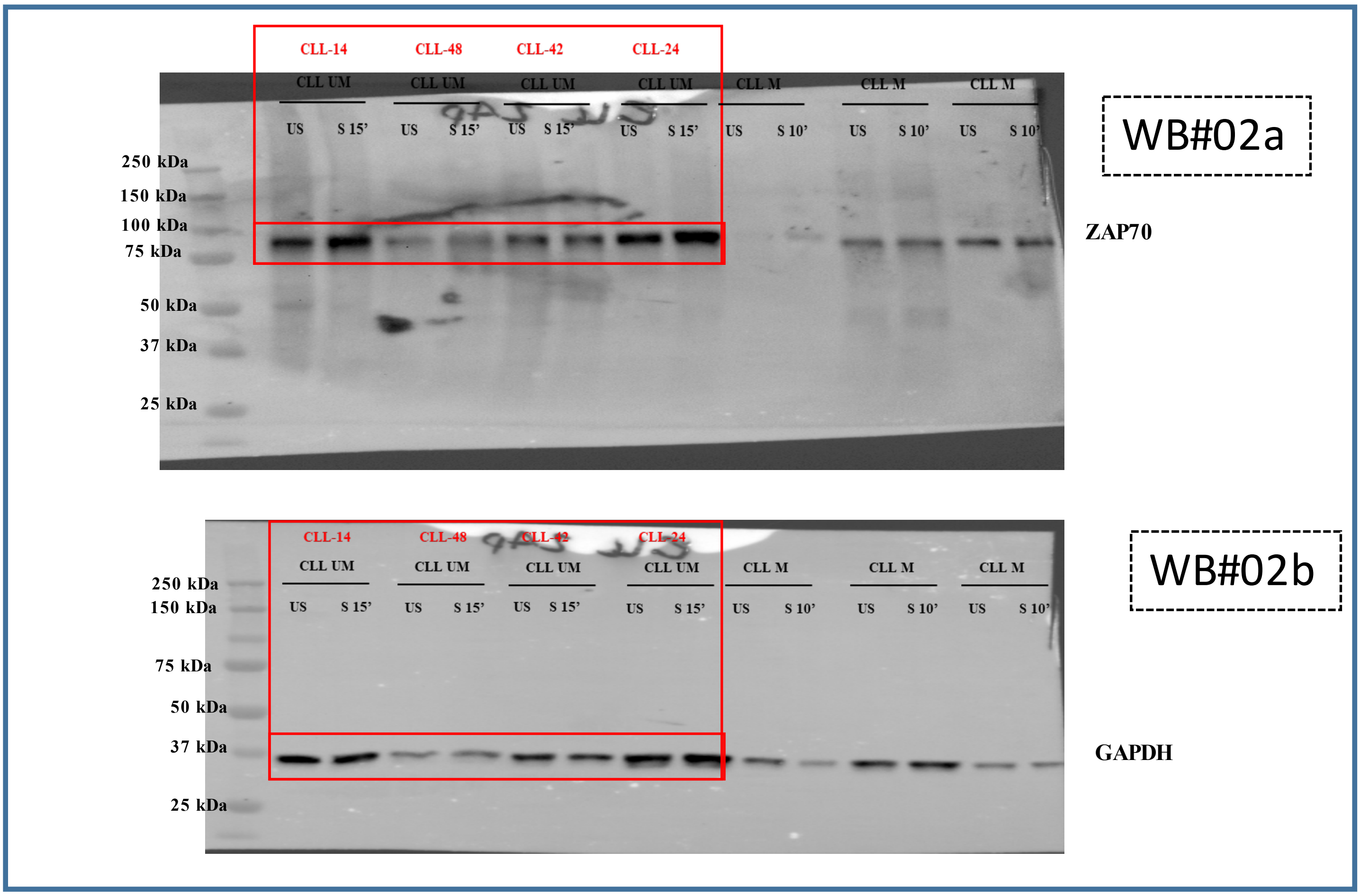
CLL-14
CLL-48
CLL-42
CLL-24
CLL UM
CLL M
US
S 15'
S 10'
250 kDa
150 kDa
100 kDa
75 kDa
50 kDa
37 kDa
25 kDa
WB#02a
ZAP70
WB#02b
GAPDH

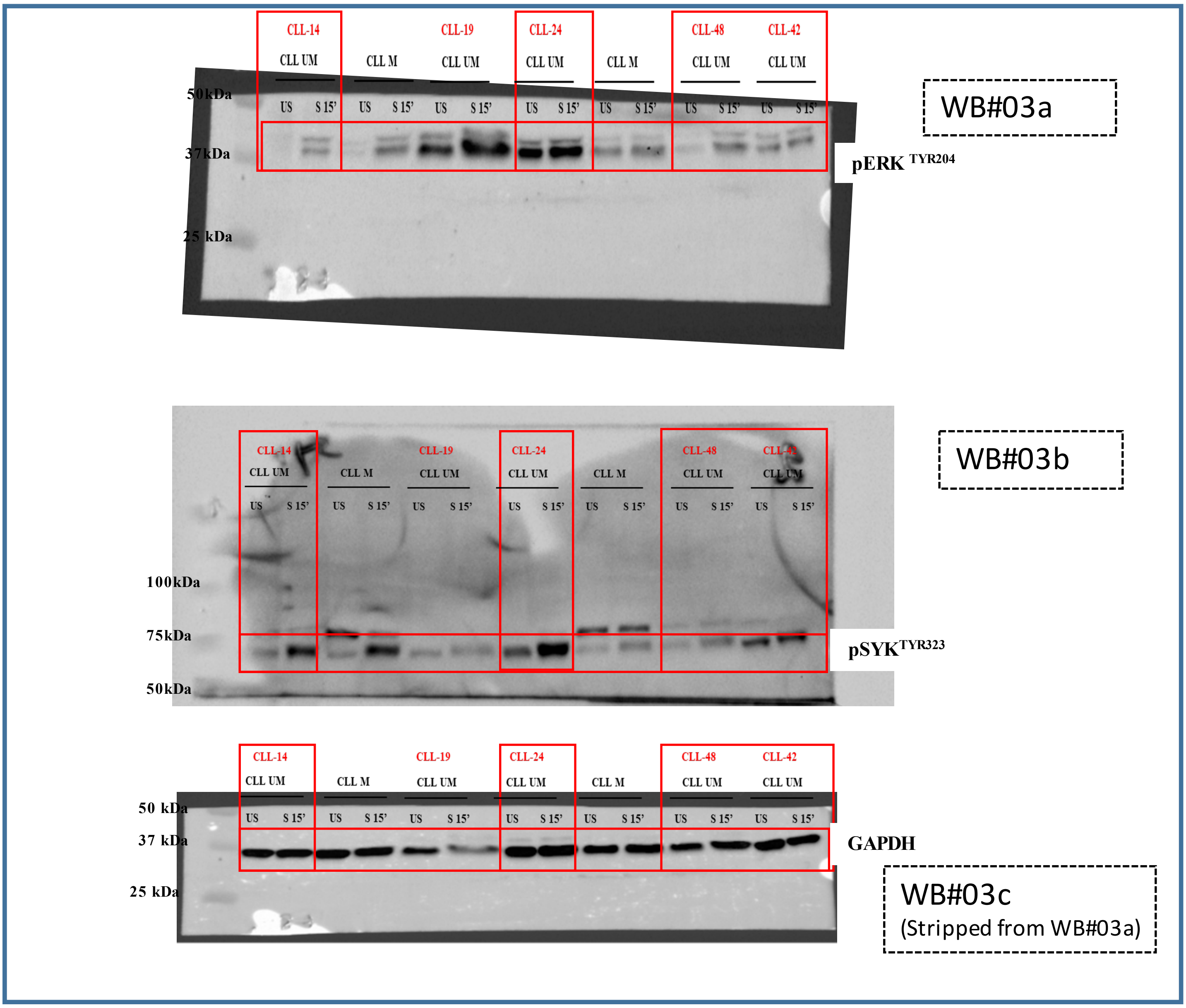
CLL-14
CLL UM
CLL M
CLL-19
CLL UM
CLL-24
CLL UM
CLL M
CLL-48
CLL UM
CLL-42
CLL UM
US S 15' US S 15' US S 15' US S 15' US S 15' US S 15' US S 15'
50kDa
37kDa
25 kDa
WB#03a
pERK TYR204
WB#03b
100kDa
75kDa
50kDa
pSYKTYR323
50 kDa
37 kDa
25 kDa
GAPDH
WB#03c
(Stripped from WB#03a)

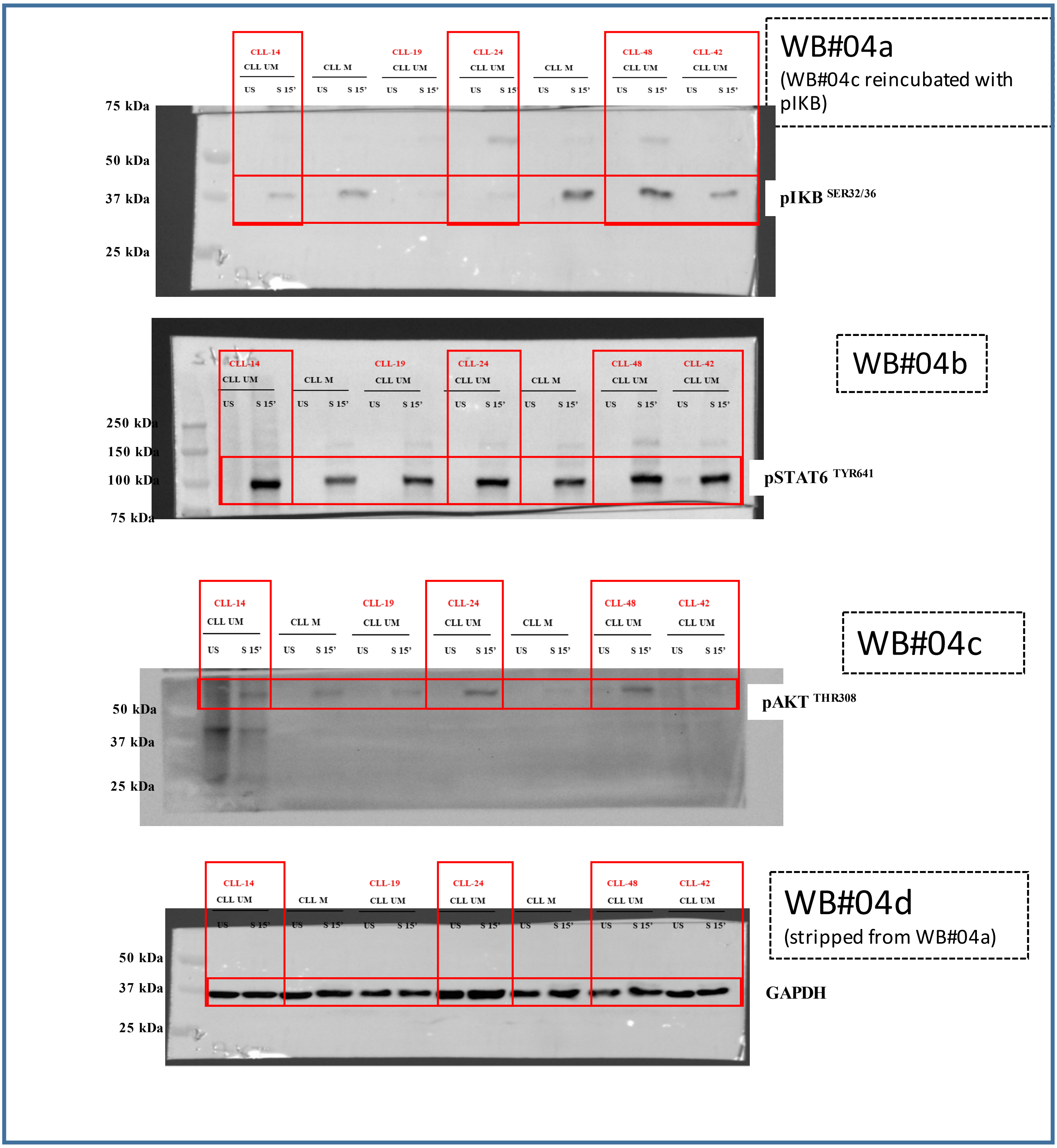
WB#04a
(WB#04c reincubated with pIKB)
pIKB SER32/36
CLL-14 CLL UM
CLL M
CLL-19 CLL UM
CLL-24 CLL UM
CLL M
CLL-48 CLL UM
CLL-42 CLL UM
US S 15'
75 kDa
50 kDa
37 kDa
25 kDa
WB#04b
pSTAT6 TYR641
250 kDa
150 kDa
100 kDa
75 kDa
WB#04c
pAKT THR308
50 kDa
37 kDa
25 kDa
WB#04d
(stripped from WB#04a)
GAPDH
50 kDa
37 kDa
25 kDa

Figure S8 B

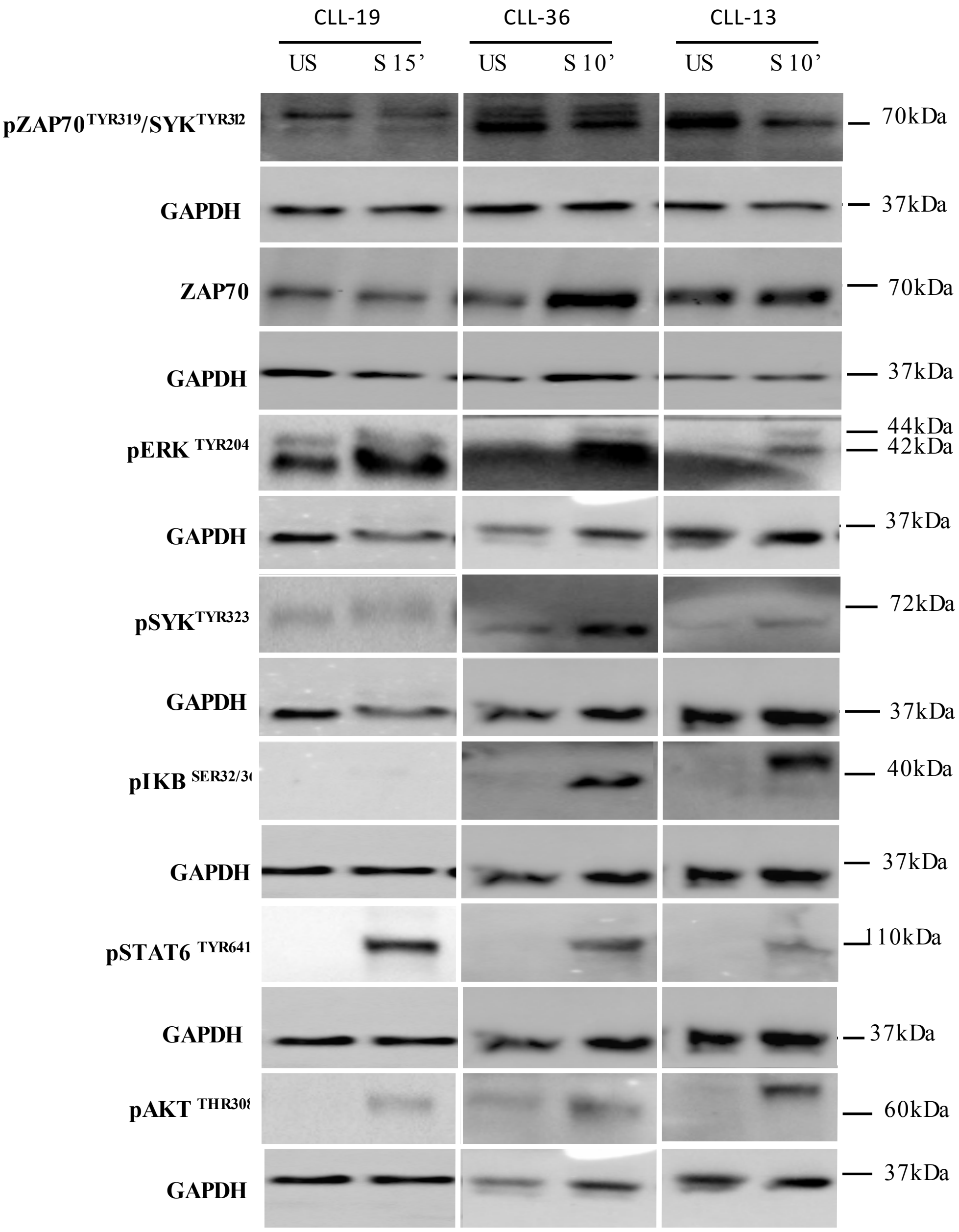
CLL-19
CLL-36
CLL-13
US
S 15'
US
S 10'
US
S 10'
pZAP70TYR319/SYKTYR352
GAPDH
ZAP70
GAPDH
pERK TYR204
GAPDH
pSYKTYR323
GAPDH
pIKB SER32/36
GAPDH
pSTAT6 TYR641
GAPDH
pAKT THR308
GAPDH
70kDa
37kDa
70kDa
37kDa
44kDa
42kDa
37kDa
72kDa
37kDa
40kDa
37kDa
110kDa
37kDa
60kDa
37kDa

Figure S8 B

	CLL-19		CLL-36		CLL-13	
	US	S 15’	US	S 10’	US	S 10’

	Blot	
pZAP70^{TYR319}/SYK^{TYR312}	WB#05a	
GAPDH	WB#05b	
ZAP70	WB#06a	
GAPDH	WB#06b	
pERK ^{TYR204}	WB#03a	WB#07a
GAPDH	WB#03c (stripped from WB#03a)	WB#07b
pSYK^{TYR323}	WB#03b	WB#08a
GAPDH	WB#03c (stripped from WB#03a)	WB#08b (Stripped from WB#08c)
pIKB $^{SER32/36}$	WB#04a (WB#04c reincubated with pIKB)	WB#08c
GAPDH	WB#04d (stripped from WB#04a)	WB#08b (Stripped from WB#08c)
pSTAT6 ^{TYR641}	WB#04b	WB#08d
GAPDH	WB#04d (stripped from WB#04a)	WB#08b (Stripped from WB#08c)
pAKT ^{THR308}	WB#04c	WB#09a
GAPDH	WB#04d (stripped from WB#04a)	WB#09b

WB#05a

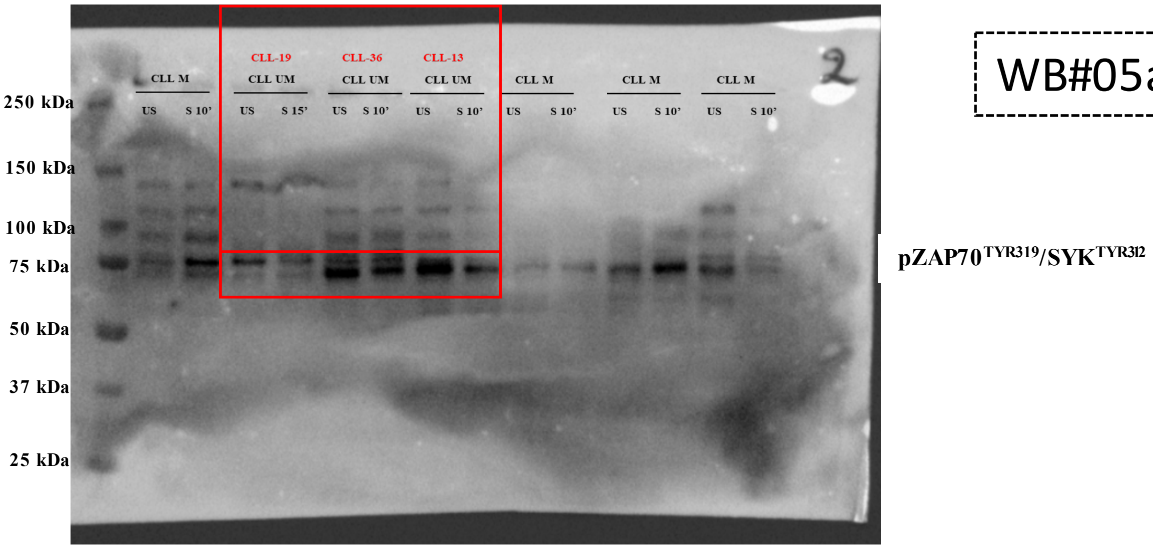

WB#05b

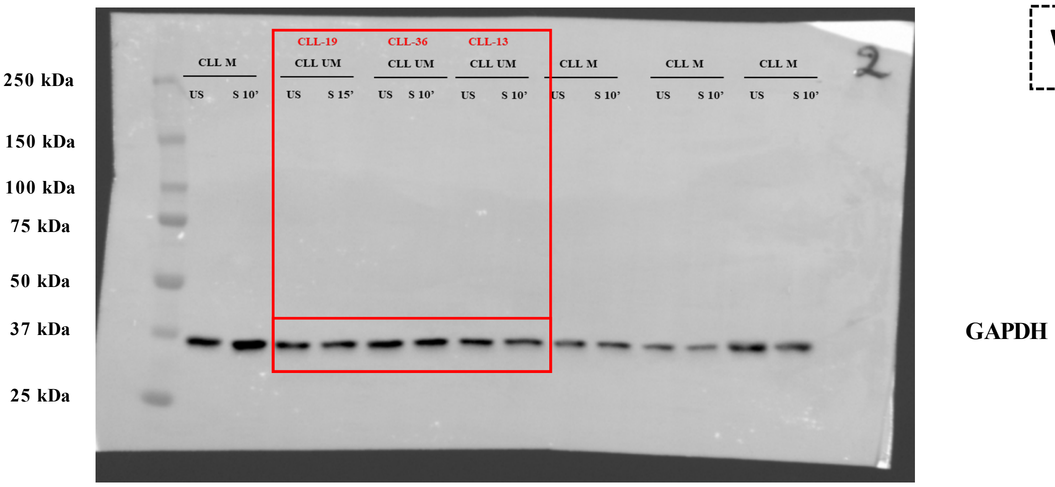

WB#06a

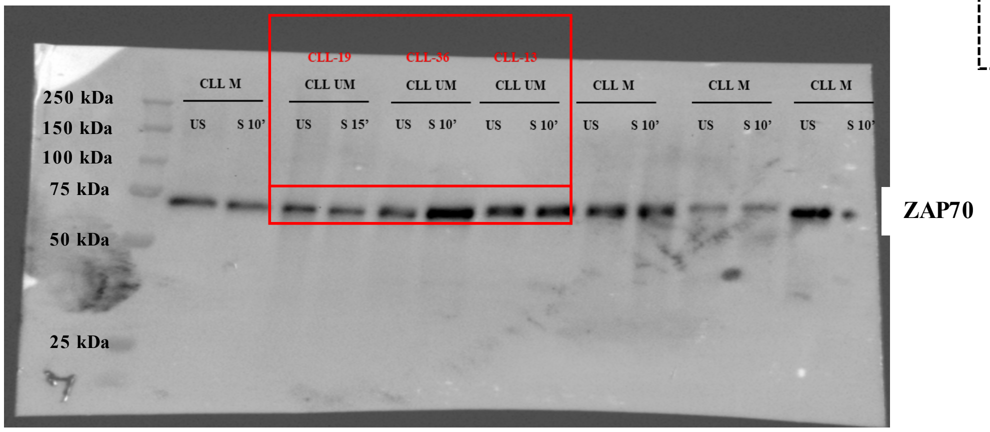

WB#06b

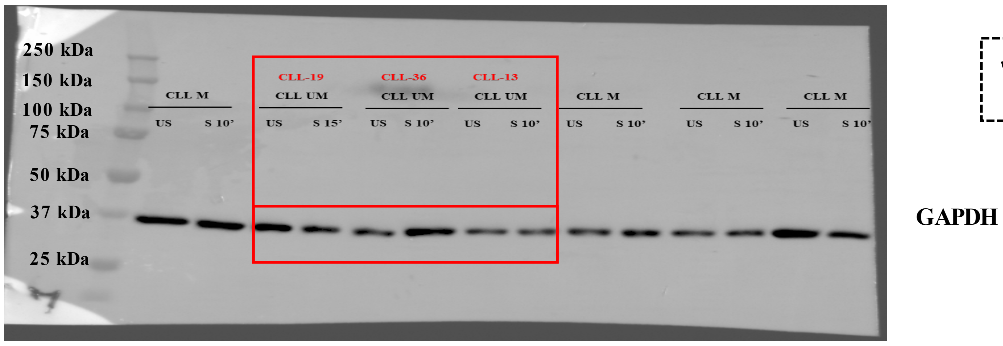

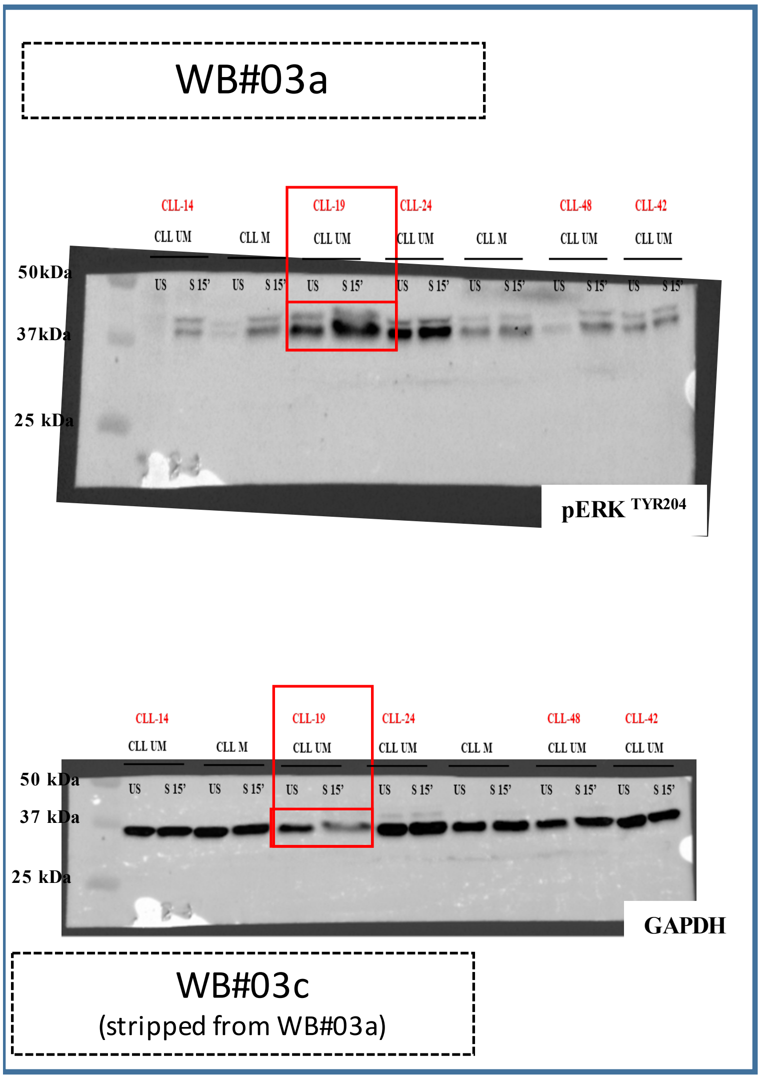
WB#03a
CLL-14
CLL UM
CLL M
CLL-19
CLL UM
CLL-24
CLL UM
CLL M
CLL-48
CLL UM
CLL-42
CLL UM
US
S 15'
50kDa
37kDa
25 kDa
pERK TYR204
CLL-14
CLL UM
CLL M
CLL-19
CLL UM
CLL-24
CLL UM
CLL M
CLL-48
CLL UM
CLL-42
CLL UM
US
S 15'
50 kDa
37 kDa
25 kDa
GAPDH
WB#03c
(stripped from WB#03a)

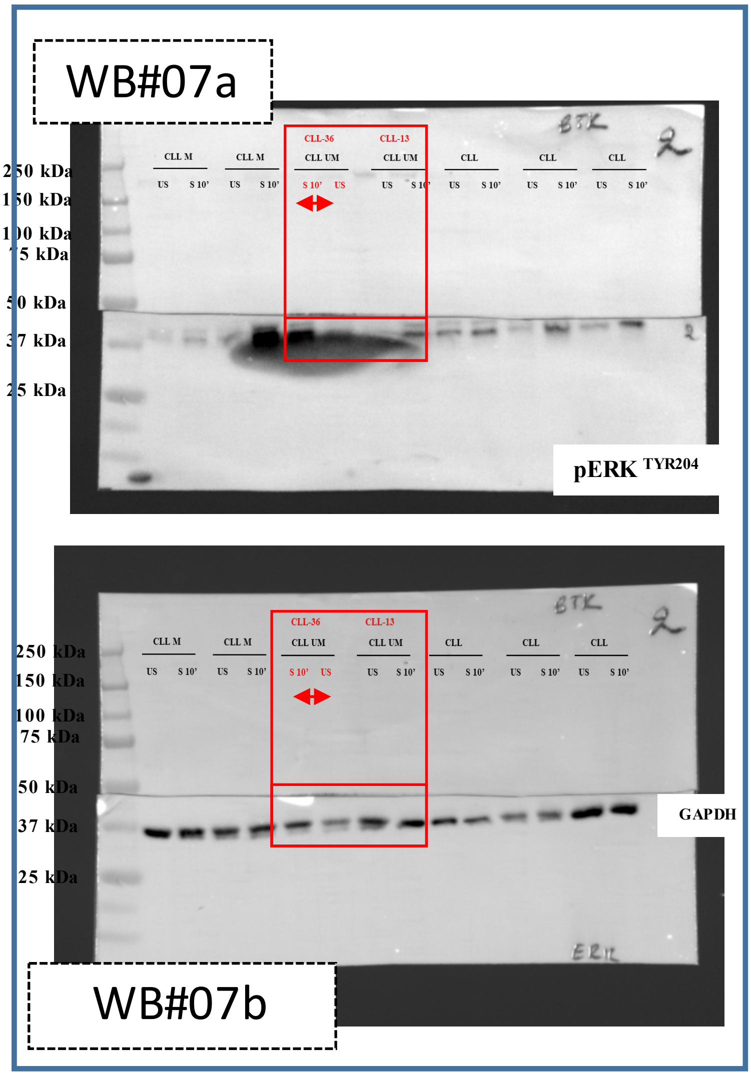
WB#07a
CLL M
CLL M
CLL-36
CLL UM
CLL-13
CLL UM
CLL
CLL
CLL
US
S 10'
250 kDa
150 kDa
100 kDa
75 kDa
50 kDa
37 kDa
25 kDa
pERK TYR204
CLL M
CLL M
CLL-36
CLL UM
CLL-13
CLL UM
CLL
CLL
CLL
US
S 10'
250 kDa
150 kDa
100 kDa
75 kDa
50 kDa
37 kDa
25 kDa
GAPDH
WB#07b

WB#03b

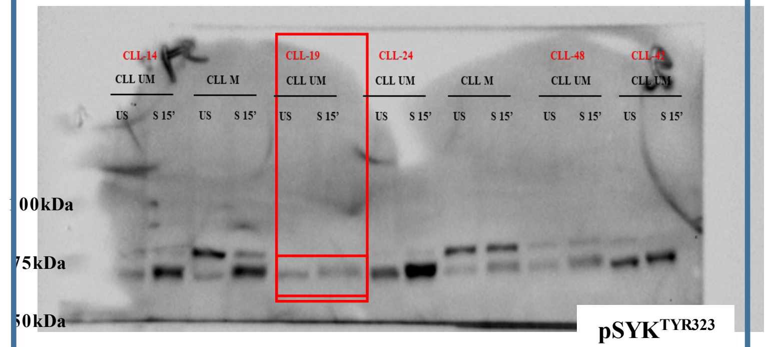

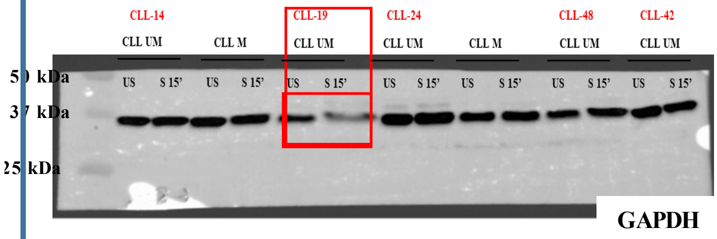

WB#03c
(stripped from WB#03a)

WB#04a
(WB#04c reincubated with pIKB)

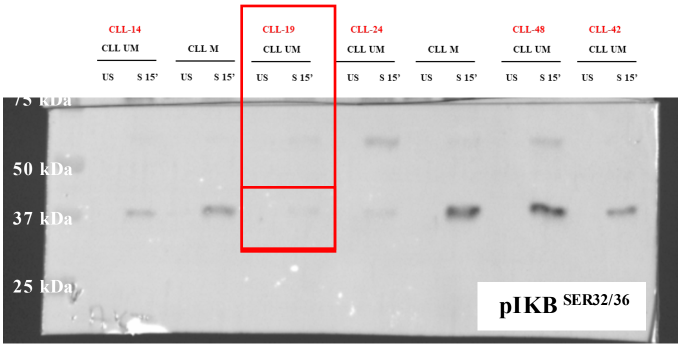

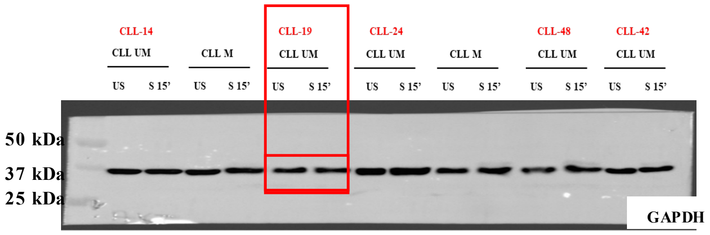

WB#04d
(stripped from WB#04a)

WB#08a

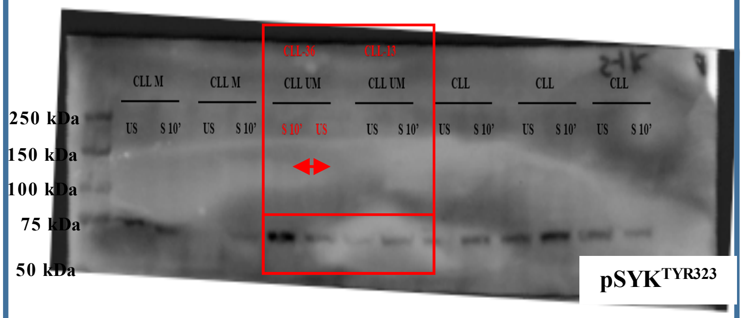

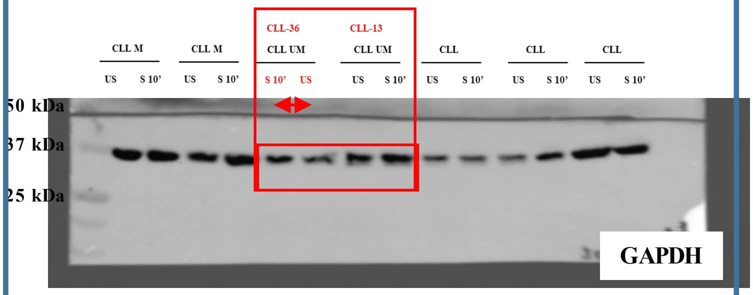

WB#08b (Stripped from WB#08c)

WB#08c

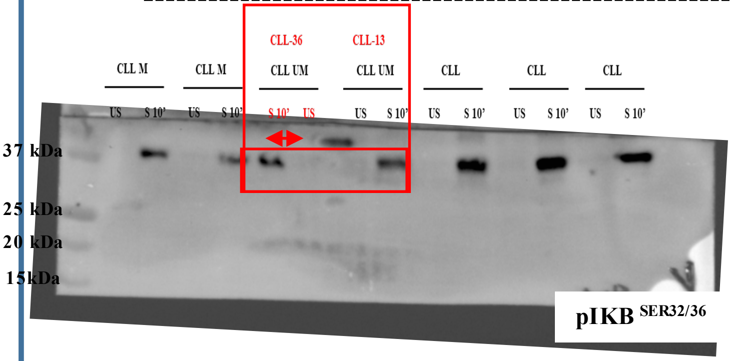

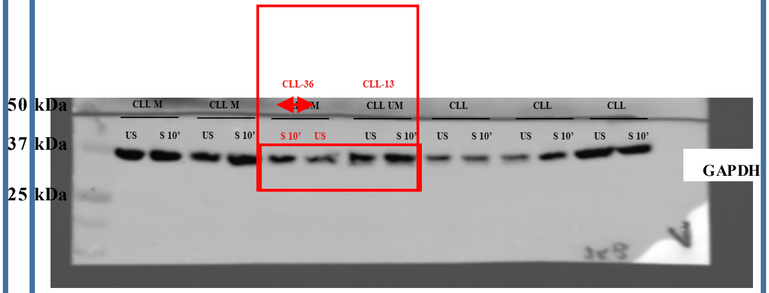

WB#08b (Stripped from WB#08c)

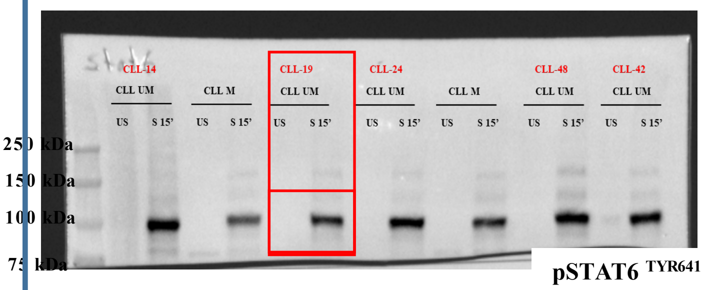

WB#04b

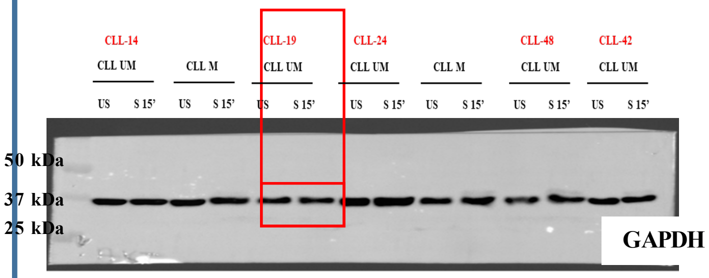

WB#04d
(stripped from WB#04a)

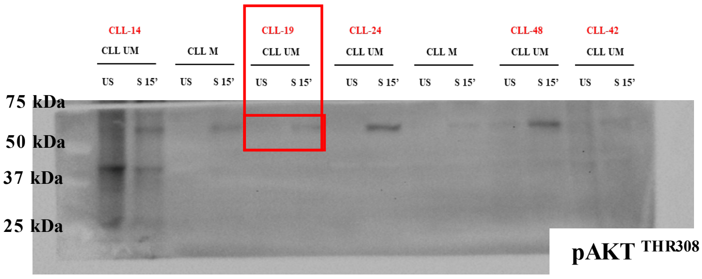

WB#04c

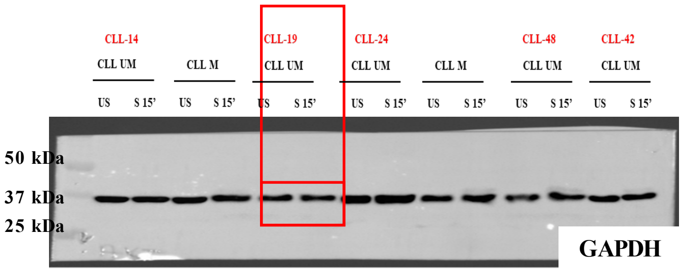

WB#04d
(stripped from WB#04a)

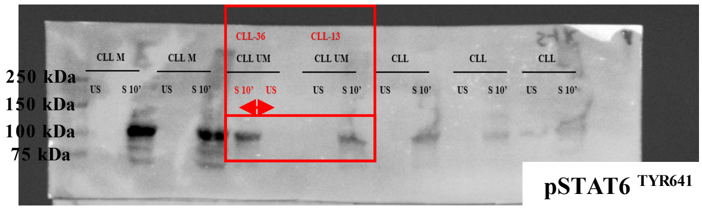

WB#08d

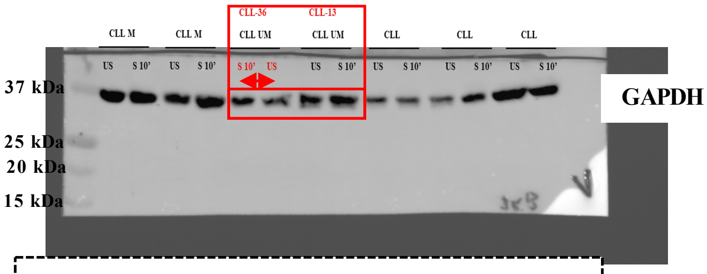

WB#08b (Stripped from WB#08c)

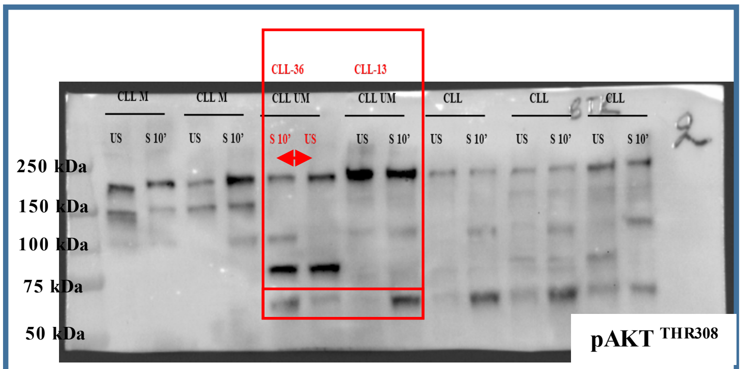

WB#09a

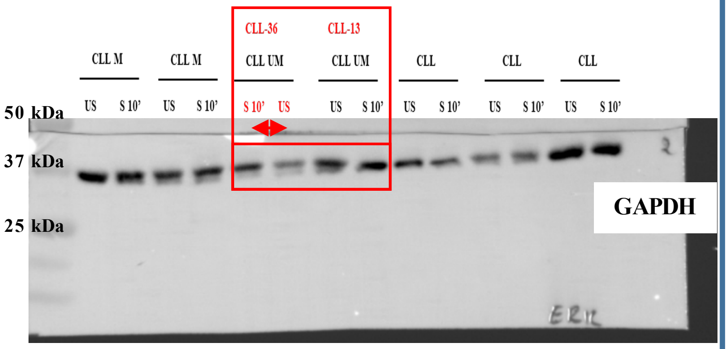

WB#09b

Figure S9 A

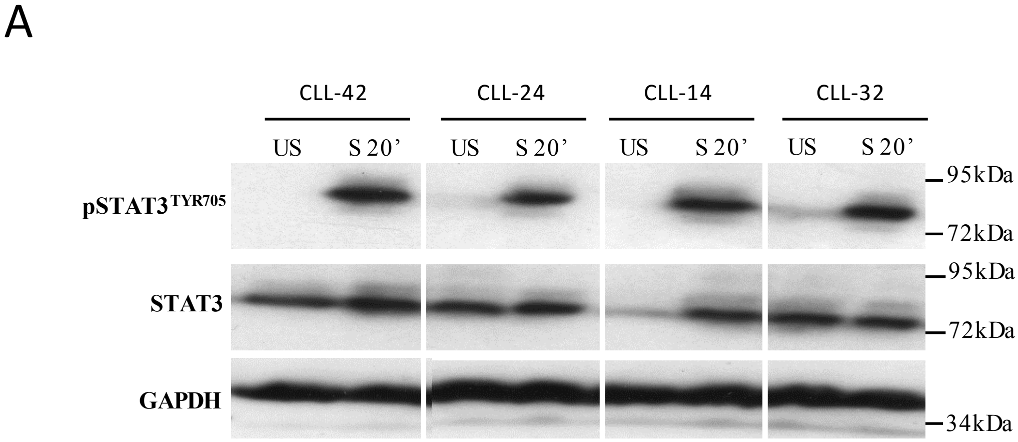
A
CLL-42
CLL-24
CLL-14
CLL-32
US
S 20'
US
S 20'
US
S 20'
US
S 20'
pSTAT3TYR705
STAT3
GAPDH
95kDa
72kDa
95kDa
72kDa
34kDa

A

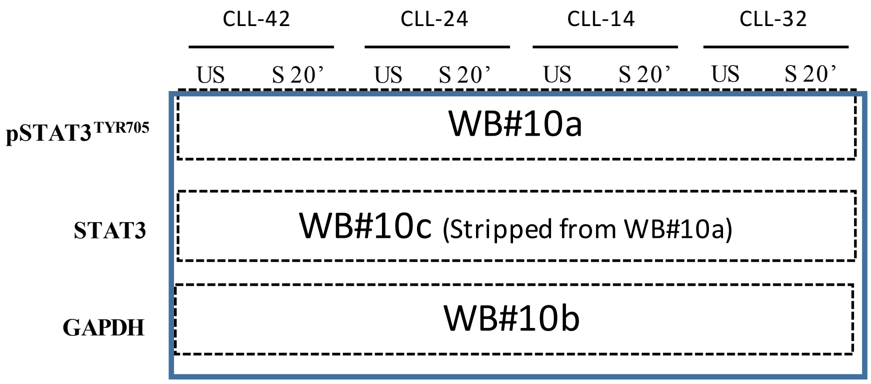
CLL-42
CLL-24
CLL-14
CLL-32
US S 20' US S 20' US S 20' US S 20'
pSTAT3 TYR705
WB#10a
STAT3
WB#10c (Stripped from WB#10a)
GAPDH
WB#10b

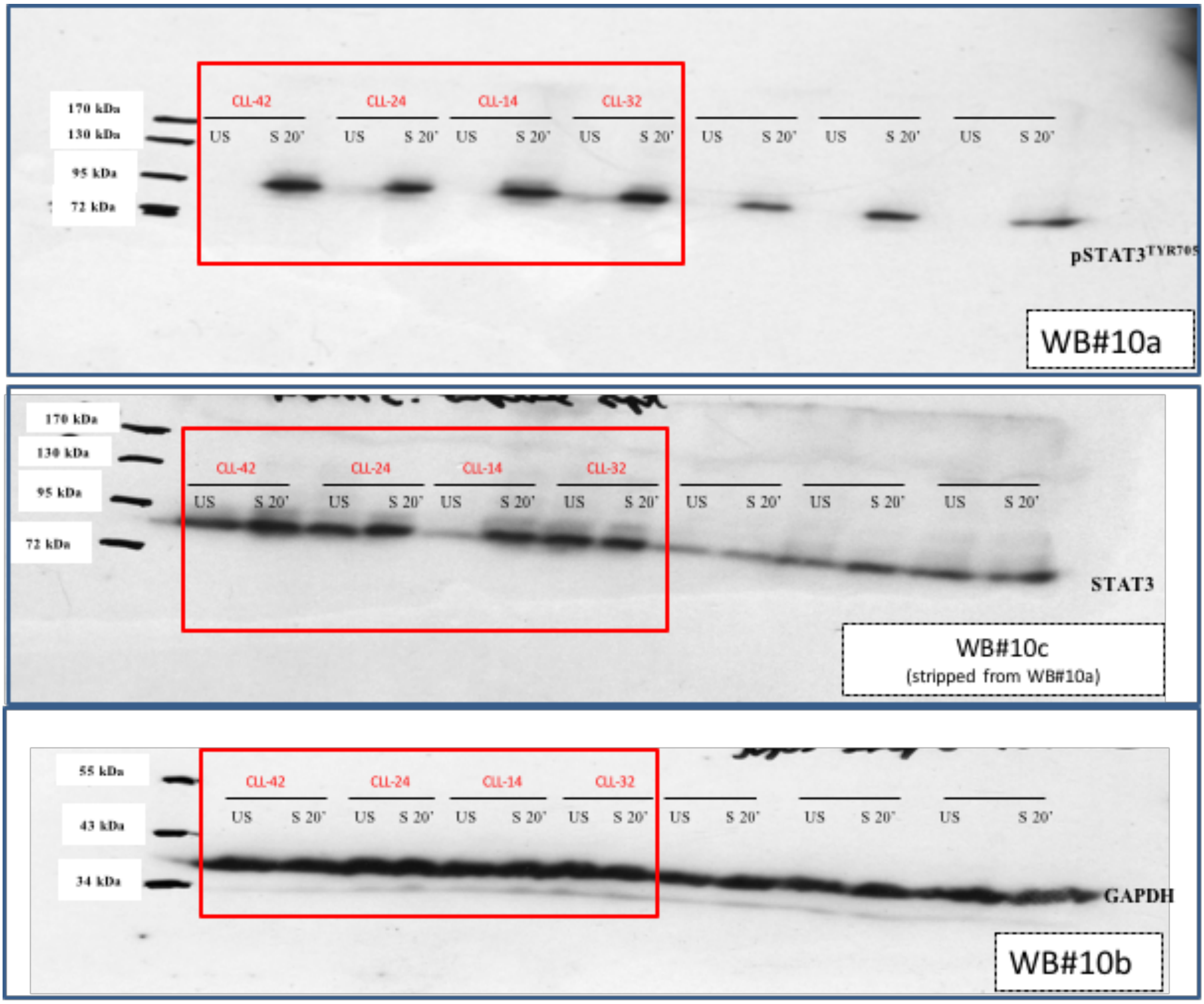
170 kDa
130 kDa
95 kDa
72 kDa
CLL-42
CLL-24
CLL-14
CLL-32
US S 20' US S 20' US S 20' US S 20' US S 20' US S 20' US S 20'
pSTAT3 TYR705
WB#10a
170 kDa
130 kDa
95 kDa
72 kDa
CLL-42
CLL-24
CLL-14
CLL-32
US S 20' US S 20' US S 20' US S 20' US S 20' US S 20' US S 20'
STAT3
WB#10c
(stripped from WB#10a)
55 kDa
43 kDa
34 kDa
CLL-42
CLL-24
CLL-14
CLL-32
US S 20' US S 20' US S 20' US S 20' US S 20' US S 20' US S 20'
GAPDH
WB#10b

Figure S9 B

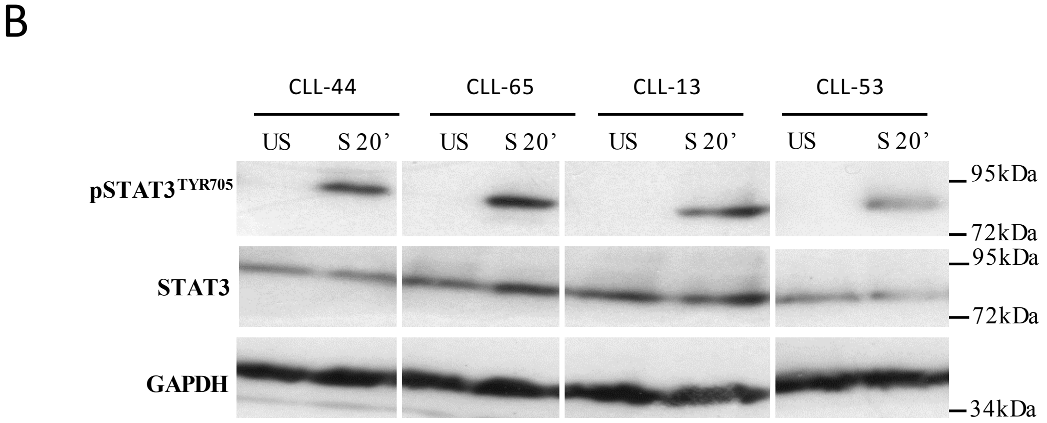
B
CLL-44
CLL-65
CLL-13
CLL-53
US
S 20'
US
S 20'
US
S 20'
US
S 20'
pSTAT3TYR705
STAT3
GAPDH
95kDa
72kDa
95kDa
72kDa
34kDa

B

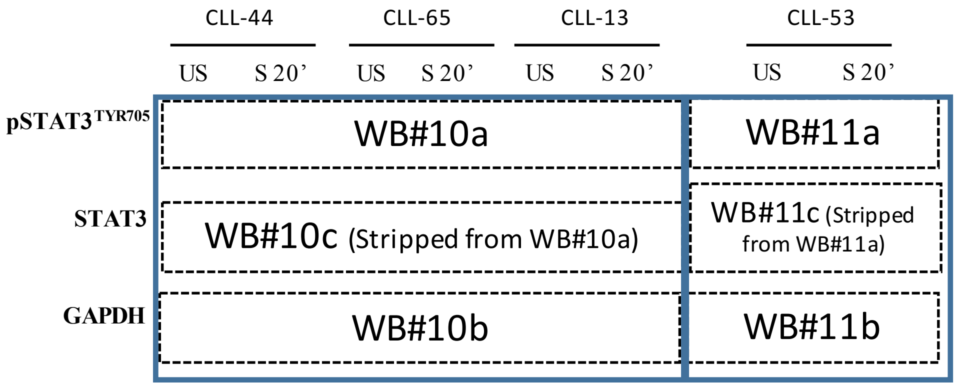
CLL-44
CLL-65
CLL-13
CLL-53
US
S 20’
pSTAT3TYR705
STAT3
GAPDH
WB#10a
WB#11a
WB#10c (Stripped from WB#10a)
WB#11c (Stripped from WB#11a)
WB#10b
WB#11b

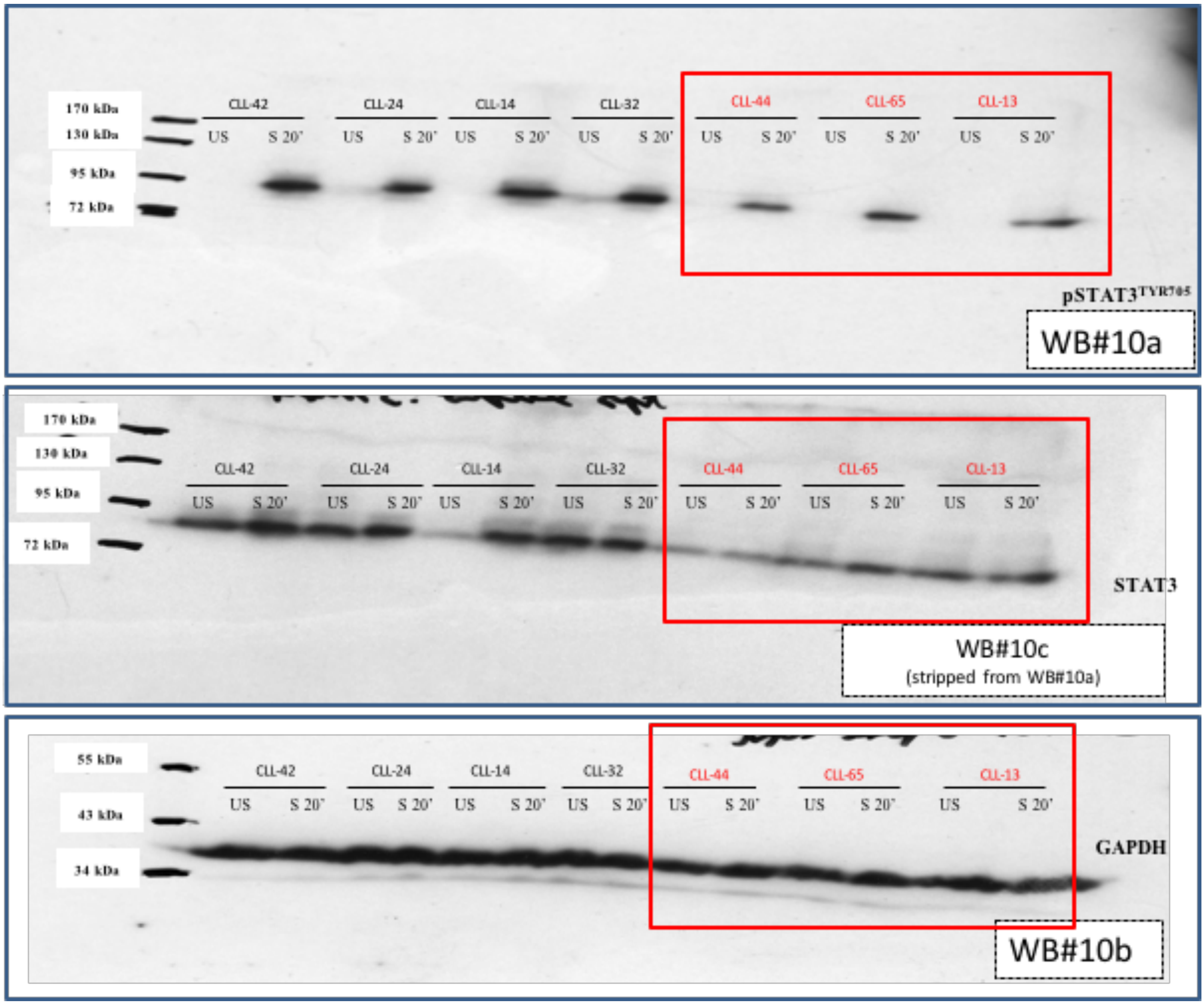
170 kDa
130 kDa
95 kDa
72 kDa
55 kDa
43 kDa
34 kDa
CLL-42
CLL-24
CLL-14
CLL-32
CLL-44
CLL-65
CLL-13
US
S 20’
pSTAT3TYR705
WB#10a
STAT3
WB#10c
(stripped from WB#10a)
GAPDH
WB#10b

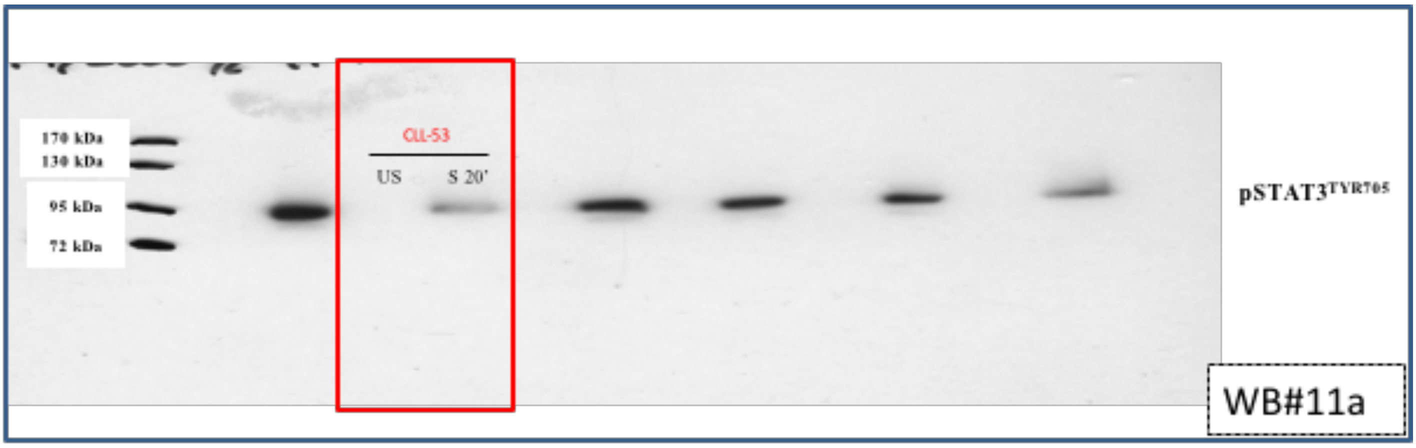
170 kDa
130 kDa
95 kDa
72 kDa
CLL-53
US
S 20'
pSTAT3TYR705
WB#11a

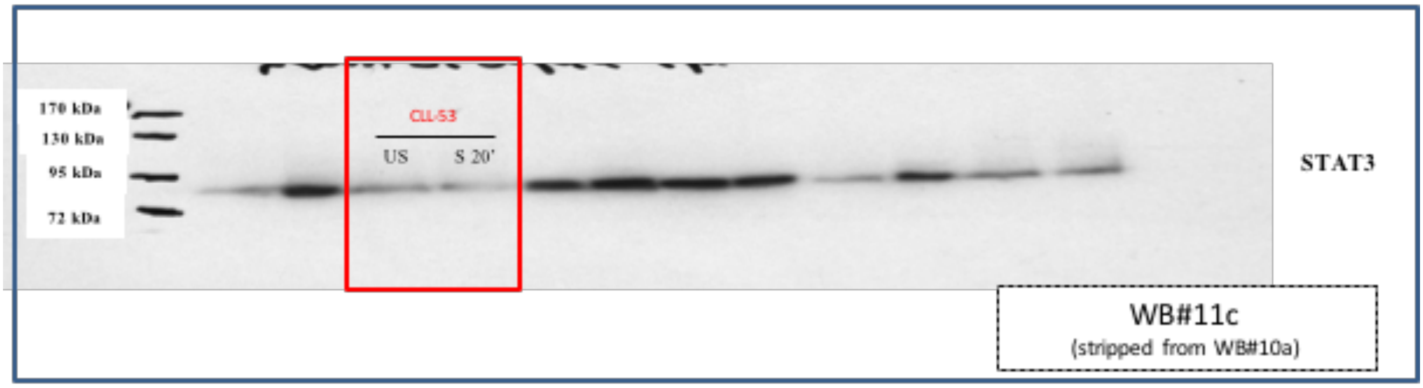
170 kDa
130 kDa
95 kDa
72 kDa
CLL-53
US
S 20'
STAT3
WB#11c
(stripped from WB#10a)

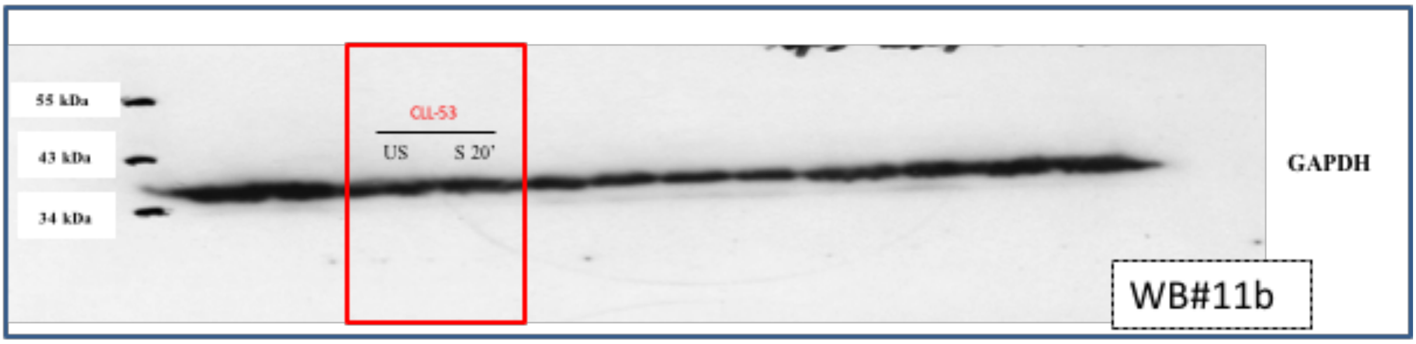
55 kDa
43 kDa
34 kDa
CLL-53
US
S 20'
GAPDH
WB#11b
